# Processing symbolic magnitude information conveyed by number words and by scalar adjectives

**DOI:** 10.1177/17470218211031158

**Published:** 2021-07-16

**Authors:** Arnold R Kochari, Herbert Schriefers

**Affiliations:** 1 FNWI, Institute for Logic, Language and Computation (ILLC), University of Amsterdam, Amsterdam, The Netherlands; 2 Donders Institute for Brain, Cognition and Behaviour, Radboud University Nijmegen, Nijmegen, The Netherlands

**Keywords:** Scalar adjectives, size congruity, numerical processing, Arabic number, verbal number

## Abstract

Humans not only process and compare magnitude information such as size, duration, and number perceptually, but they also communicate about these properties using language. In this respect, a relevant class of lexical items are so-called *scalar adjectives* like “big,” “long,” “loud,” and so on which refer to magnitude information. It has been proposed that humans use an amodal and abstract representation format shared by different dimensions, called *the generalised magnitude system* (GMS). In this paper, we test the hypothesis that scalar adjectives are symbolic references to GMS representations, and, therefore, GMS gets involved in processing their meaning. Previously, a parallel hypothesis on the relation between number symbols and GMS representations has been tested with the size congruity paradigm. The results of these experiments showed interference between the processing of number symbols and the processing of physical (font-) size. In the first three experiments of the present study (total *N* = 150), we used the size congruity paradigm and the same/different task to look at the potential interaction between physical size magnitude and numerical magnitude expressed by number words. In the subsequent three experiments (total *N* = 149), we looked at a parallel potential interaction between physical size magnitude and scalar adjective meaning. In the size congruity paradigm, we observed interference between the processing of the numerical value of number words and the meaning of scalar adjectives, on the one hand, and physical (font-) size, on the other hand, when participants had to judge the number words or the adjectives (while ignoring physical size). No interference was obtained for the reverse situation, i.e., when participants judged the physical font size (while ignoring numerical value or meaning). The results of the same/different task for both number words and scalar adjectives strongly suggested that the interference that was observed in the size congruity paradigm was likely due to a response conflict at the decision stage of processing rather than due to the recruitment of GMS representations. Taken together, it can be concluded that the size congruity paradigm does not provide evidence in support the hypothesis that GMS representations are used in the processing of number words or scalar adjectives. Nonetheless, the hypothesis we put forward about scalar adjectives is still is a promising potential line of research. We make a number of suggestions for how this hypothesis can be explored in future studies.

## Introduction

A lot of research has been devoted to the question how the human cognitive system estimates and compares magnitudes such as size, length, quantity, loudness, duration, and so on from the perceptual input. Based on the accumulated evidence, some researchers postulated the existence of a generalised (analog) magnitude system (GMS; Cantlon et al., 2009; Lourenco, 2015; Walsh, 2003). Under one such proposal, after our perceptual system takes up the information from the outside world (e.g., in visual modality about length, size or quantity), this information is transformed into an abstract, amodal representation format in which the comparison of magnitudes can be performed. For example, when comparing the lengths of lines, information about length will be mapped onto the same cognitive system as the one used for comparing quantities of objects in two sets, the duration of two auditory signals, and so on.

The ability to estimate and compare magnitudes is not only used to perceptually navigate in the world around us, but information about such magnitudes is also communicated to others. At the same time, when others communicate information about magnitudes to us, we need to interpret this information. In this respect, an interesting class of lexical items are so-called *scalar adjectives* (also referred to as *vague* or *gradable*) such as “tall,” “short,” “long,” “big,” “loud,” and so on. Interestingly, the meaning of all these adjectives shares a set of features with the format in which magnitudes are thought to be represented in GMS. In this paper, we therefore put forward and test the hypothesis that scalar adjectives are symbolic references to GMS representations, and, thus, when processing their meaning, our language comprehension system makes use of GMS representations.

We present a set of six experiments carried out with the goal to investigate the involvement of GMS in the processing of number words (Experiments 1a, 1b) and scalar adjectives (Experiments 2a, 2b). These experiments make use of the size congruity paradigm, and Experiments 1c and 2c will therefore use a new experimental approach to address the question whether the congruity effects obtained with this paradigm are due to representational overlap/shared representations or due to response conflicts at a response decision stage.

### Generalised (analog) magnitude representation system

Let us start with a note on terminology: we will use the word *magnitude* to refer to values along any continuous dimension (e.g., size magnitude, length magnitude, numerical magnitude), and we will use the word *quantity* to refer specifically to numerical magnitude (i.e., the number of distinct individual elements). Furthermore, we will use the word *nonsymbolic magnitude* to refer to magnitudes extracted from perceptual input (e.g., a visually presented array of dots or an auditory sequence of tones) as opposed to *symbolic* references to magnitude such as number symbols and scalar adjectives (discussed in the remaining sections of the introduction).

Most of the work on magnitude processing has been done on numerical magnitude processing, so we start by introducing what is known about numerical magnitude processing. When receiving and evaluating numerical magnitude information from perceptual input, our cognitive system makes an approximation of magnitude (rather than providing us with a precise value), and it has a limited sensitivity with which it can do so. For example, when extracting a quantity from a visual scene, we are able to successfully distinguish a set of 15 dots from a set of 30 dots, but not 28 dots from 30 dots. Performance with nonsymbolic quantities in terms of accuracy and reaction times is dependent on the ratio between the two quantities to be compared such that larger ratios (i.e., larger relative difference in magnitude) lead to faster and more accurate responses than smaller ratios (i.e., smaller relative difference in magnitude; e.g., Buckley & Gillman, 1974; Feigenson et al., 2004; Halberda & Feigenson, 2008; Pica et al., 2004 this is consistent with Weber’s law, see e.g., Bar et al., 2019 for a recent discussion). Such performance has been suggested to reflect the operation of the so-called Approximate Number System (ANS) which is thought to be an evolutionary old system shared with other animals (Barth et al., 2003; Dehaene, 1997; Feigenson et al., 2004; Gallistel & Gelman, 2000; Halberda & Feigenson, 2008). Numerical magnitude representations in ANS are thought to be continuous (or *analog*) distributions around a point (similar to a Gaussian distribution) which overlap with neighbouring distributions. Two alternative accounts have been proposed—either the spread of the distributions around points increases with increasing quantities or the spread of distributions is same for different quantities but the quantities are logarithmically compressed (e.g., Bar et al., 2019; Dehaene et al., 2008; Feigenson et al., 2004; Gallistel & Gelman, 1992; Merten & Nieder, 2008; Nieder, 2016; see also recent alternatives to ANS which propose that information about magnitude of other dimensions is used to infer quantity—(Anobile et al., 2016; Gebuis et al., 2016; Leibovich et al., 2017).

In the same way as we can approximate a quantity from perceptual input, we can also make such approximations on the length of a line, the duration of an event, the size of an object, and so on. These approximate judgements are also limited in precision and, interestingly, are also ratio-dependent (see e.g., Table 1 in Cohen Kadosh et al., 2008 for examples). It has been suggested that there is a single shared underlying system for representing and processing perceived magnitudes in various continuous dimensions (including numerical magnitude). This system has been referred to as the *GMS* (Gallistel & Gelman, 2000; Walsh, 2003, 2015) and is usually conceived of as a generalised version of ANS. Magnitudes in such a system are assumed to be represented in the same way as numerical magnitudes are represented in the ANS—as continuous (analog) distributions around a point which overlap with neighbouring values and have increasing uncertainty with increasing values (see the *online Supplementary Material A* for a short review of discussion around existence of GMS; because this is a large field of study of its own, we will not discuss the details in this manuscript; for the purpose of our project, we make the assumption described below).

**Table 1.** Overview of materials, tasks and goals in each presented experiment.

Experiment	Materials	Task	Goal
1a	Number words	Comparison task: choose larger/smaller numerical value	Investigate previously reported size congruity effect with number words while controlling for discriminability and variability
1b		Comparison task: choose larger/smaller font size	Investigate previously unobserved size congruity effect with number words while controlling for discriminability and variability
1c		“Same”/“different” judgement	Disentangle representational overlap and decisional stage conflict accounts of the size congruity effect
2a	Scalar adjectives	Comparison task: choose larger/smaller meaning	Investigate size congruity effect with scalar adjectives
2b		Comparison task: choose larger/smaller font size	Investigate size congruity effect with scalar adjectives
2c		“Same”/“different” judgement	Disentangle representational overlap and decisional stage conflict accounts of the size congruity effect

In the present research project, we assume that there exists a GMS-like mechanism that is responsible for computing relative magnitude in various dimensions. This shared mechanism computes relative magnitudes when we are comparing, for example, numerical magnitudes, size magnitudes, length magnitudes, duration magnitudes, and so on from *perceptual* input.

### Processing number symbols

Our cognitive system can receive and process quantity information not only from perceptual input, but also *symbolically*—e.g., using Arabic digits (“3,” “5”) or number words (“three,” “five”). Note that number symbols refer to exact, discrete quantities whereas nonsymbolic quantity is perceived in a continuous format, i.e., without sharp boundaries (e.g., Bar et al., 2019; Leibovich et al., 2013).

To what extent number symbols (e.g., an Arabic digit) are represented by the same cognitive system or recruit the same processing mechanisms as perceptual, nonsymbolic quantity remains a matter of a debate (see e.g., Nieder, 2016; Piazza & Eger, 2016; Sokolowski & Ansari, 2016; Wilkey & Ansari, 2019 for extensive reviews). It has been suggested that — as a cultural invention — number symbols use (or *recycle*) the evolutionary older ANS-type of representations of quantity (Dehaene & Cohen, 2007). To explore this possibility, one line of research looked at whether parallel behavioural effects can be observed for both number symbols and nonsymbolic quantity which would suggest shared representations or at least shared processing mechanisms. Parallel ratio-based performance with both symbolic and nonsymbolic numerical magnitudes has been reported, for example, in quantity comparison tasks (e.g., Dehaene, 2007; Dehaene & Akhavein, 1995; Moyer & Landauer, 1967), though the interpretation of these effects as supporting shared cognitive systems for the two formats has been contested (e.g., Kojouharova & Krajcsi, 2020; Krajcsi et al., 2018; Verguts et al., 2005). Further evidence comes from matching and priming paradigms that showed that number symbols closer to each other in terms of their numerical magnitude seem to have more overlap in their representations than number symbols further away from each other, as would be expected if they recruit the nonsymbolic, ANS representations (e.g., Defever et al., 2011; Reynvoet et al., 2009; Sasanguie et al., 2011; Van Opstal et al., 2008; Van Opstal & Verguts, 2011), but there is again some counter-evidence (Roggeman et al., 2007; Sasanguie et al., 2017).

Brain imaging studies report ratio-dependent changes in the amount of BOLD signal in the intraparietal cortex when processing both symbolic and nonsymbolic quantities (e.g., Cantlon et al., 2006; Goffin et al., 2019; He et al., 2015; Holloway et al., 2012; Piazza et al., 2004; Vogel et al., 2017). On the other hand, recent studies making use of activation pattern analysis techniques (e.g., representational similarity analysis [RSA]) report that the pattern of voxelwise activity correlations in the intraparietal cortex and some other areas corresponded to overlapping analog representations for nonsymbolically presented quantities, but not for number symbols (Bulthé et al., 2014, 2015; Lyons et al., 2015; Lyons & Beilock, 2018). We do not review discussions around these findings here as it is a separate field of research by itself (see Eger, 2016; Wilkey & Ansari, 2019, for recent reviews).

Given the mixed evidence, deciding whether and to what extent symbolic and nonsymbolic quantity have overlapping representations requires further research. The present project does not aim to resolve this issue. Rather, the central goal of the present project is to apply an existing paradigm that has previously been used to investigate the potential relationship between number symbols and nonsymbolic quantity as a starting point for investigation of processing scalar adjectives (introduced in the next subsection). However, in doing so, as a first step we also use this experimental paradigm to number words. Thus, the results of the present study will partially also contribute to the number symbol processing research.

Above, we discussed the proposal that there exists a single shared cognitive system for processing magnitudes from perceptual input along various dimensions, GMS. If one assumes that a GMS-like cognitive system exists, and if number symbol representations are indeed partially shared with nonsymbolic numerical magnitude representations, then number symbol representations should also be partially shared with nonsymbolic magnitude representations in other dimensions. A number of studies has presented evidence for this relationship in the past. As we discuss below in detail, this has been more convincingly demonstrated in case of Arabic digits than in case of number word processing. In the present study, we test this prediction in case of specifically number words (as references to numerical magnitude) and size magnitude (Experiments 1a-1c). Before we do that, let us introduce the parallel hypothesis regarding scalar adjectives, i.e., the type of magnitude information carrying elements that we are primarily interested in in this project.

### Scalar adjectives

Similarly to numerical magnitude, magnitude information along other dimensions can also be conveyed symbolically. In natural language, we can describe an object’s magnitude along a particular dimension using adjectives such as “big” and “small,” “long,” and “short,” “loud,” and “quiet,” etc. For example, we can describe a new TV at our neighbours’ house as being “big” or this morning’s weekly work meeting as lasting “long.” This class of adjectives is referred to as *scalar adjectives* (or sometimes *vague* or *gradable adjectives*; see e.g., Frazier et al., 2008; Solt, 2015; van Rooij, 2011 for reviews).

Scalar adjectives seem to possess some of the properties of the GMS representation format (earlier observed by Fults, 2011). First, we can use adjectives like “tall” to describe quite different heights—e.g., that of buildings, trees or people. This suggests that these adjectives are flexible in their magnitude reference and what seems to matter for applicability of these adjectives is relative magnitude in a given context, not the absolute value. This property is consistent with our suggestion that they are referring to GMS-like representations because there too, what matters in comparison are relative rather than absolute values.

The second relevant property is that these adjectives lack sharp boundaries that determine when they do and when they do not apply as descriptions of a particular magnitude. For example, there is no one specific height that we refer to as being “tall,” not even if we talk about something specific, e.g., “a tall building.” Furthermore, if we earlier referred to some building as being “tall” and now see a different building that is only slightly shorter, then we would have to admit that “tall” also applies to this slightly shorter building, and in this situation it is impossible to come up with a strict criterion for when a building is not tall anymore when we take small steps (relative to the absolute magnitude). Thus, these symbolic magnitudes are like nonsymbolic magnitudes—they are represented in a continuous format with no strict boundaries and small differences between magnitudes are not perceptible. Similarly, we can count the number of floors of a building or measure the size of an object using exact numbers, but we would still not know when “tall” and “big” exactly do and do not apply. This once again demonstrates that these adjectives do not refer to or involve discrete magnitudes in interpretation.

Scalar adjectives have been a subject of extensive research within philosophy of language and semantics, but received relatively little attention in psycholinguistics. Researchers in psycholinguistics may be familiar with scalar adjectives from the line of research started by the work of Sedivy and colleagues (Sedivy et al., 1999) looking into whether participants take into account the meaning of the scalar adjectives immediately as they hear them (i.e., incrementally) and interpret them in relation to the contrast between objects that they see simultaneously on the display. A different research line used the fact that the meaning of scalar adjectives depends on the noun that it combines with to investigate the timing and the neural correlates of semantic composition of minimal adjective-noun phrases (Kochari et al., 2021; Ziegler & Pylkkänen, 2016).

Let us now turn to the present research project. Above, we discussed the GMS. We assume that it is recruited for processing nonsymbolic magnitudes in different dimensions. Furthermore, we proposed that scalar adjectives in language can be seen as symbolic references to the magnitudes that they refer to, and that they do so. Departing from these two observations, in the present study we ask whether such GMS-like representations are recruited in the processing of scalar adjectives just as it has been shown by a number of studies on number symbols, on nonsymbolic numerical magnitude and on other non-numerical magnitudes. We suggest that our language processing system makes use of the GMS representations during the retrieval of the meaning of scalar adjectives and the construction of a mental model of the communicated information. Thus, for example, in order to understand a phrase like “a long meeting,” we make use of the GMS in order to imagine this meeting being longer than some other meeting we experienced. We test this hypothesis by investigating whether we can observe an interference between magnitude information conveyed perceptually and magnitude information extracted when processing scalar adjectives (Experiments 2a-2c).

Under our hypothesis, the processing of scalar adjectives should recruit GMS-like representations in a way similar to the processing of number symbols. At the same time, we know that number symbols (at least in principle) refer to exact values whereas scalar adjectives refer to imprecise values. Therefore, it is possible that the precision of the retrieved magnitudes is higher for number symbols than for scalar adjectives. In this case different quantitative predictions could be made for scalar adjectives and number symbols. We did not address this potential difference in the current study. Instead, we only addressed the question of the same general direction of (congruity-) effects in case of scalar adjectives and number symbols.

We now turn to the discussion of the experimental paradigm that we use.

### Size congruity effect as an indicator of shared representations across different magnitude dimensions

A classical experimental set-up that has been used to demonstrate interference of symbolic and nonsymbolic magnitude information from different dimensions is the number size congruity paradigm (Besner & Coltheart, 1979; Henik & Tzelgov, 1982 for recent studies using this paradigm see e.g., (Arend & Henik, 2015; Cohen Kadosh et al., 2008; Gabay et al., 2013; Leibovich et al., 2013; Santens & Verguts, 2011). In this paradigm which is similar to the Stroop task, participants are typically presented with two Arabic digits side by side on a screen. They are asked to decide which one has the larger or the smaller numerical magnitude. The relative font size of the two digits (which is irrelevant for the task) is manipulated such that it either agrees with the numerical magnitude information (**5** 3; congruent condition) or is in conflict with it (5 **3**, incongruent condition). In the second version of the paradigm, the dimensions are reversed—participants have to ignore the numerical magnitude and instead decide which of the two presented digits is of physically larger or smaller font size. In this version of the task, again, numerical information either agrees or is in conflict with size information. A robust congruity effect has been observed in both versions: reaction times are shorter in the congruent condition than in the incongruent condition.

The size congruity effect has been interpreted as evidence for two aspects of magnitude processing: automaticity of computation of numerical and physical size magnitude (e.g., Dadon & Henik, 2017; Henik & Tzelgov, 1982; Pansky & Algom, 1999; Tzelgov et al., 1992) and shared representations underlying numerical and size magnitudes (e.g., Arend & Henik, 2015; Cohen Kadosh et al., 2008; Schwarz & Heinze, 1998). Let us consider the first point. The size congruity effect shows that both physical and numerical magnitude are able to interfere with performance even though they are task-irrelevant. Because information in the task-irrelevant dimension could not be completely ignored in this task, it has been suggested that physical size and numerical magnitude are automatically computed (in case of physical size) or retrieved (in case of numerical magnitude). To what extent are these computations automatic? On a strong automaticity account, no general processing resources would be required for computation or retrieval of magnitude. However, the congruity effect has been shown to be modulated (but not eliminated) by the discriminability of physical sizes and digit pairs as well as to some extent by practice and motivation, so strong automaticity can be ruled out (Algom et al., 1996; Dadon & Henik, 2017; Pansky & Algom, 1999). Instead, the size and numerical magnitude computations seem to be automatic in the sense that activation of magnitude representations is obligatory (at least in the size congruity paradigm), but does require processing resources, and cognitive control can be exerted to some extent (Dadon & Henik, 2017; Pansky & Algom, 1999).^1^

The size congruity effect has also been interpreted as evidence in favour of shared representations of numerical magnitude and physical size magnitude (but see Risko et al., 2013; Santens & Verguts, 2011 for alternative interpretations, to be discussed below). Specifically, it has been proposed that both the retrieved numerical magnitude of a digit and its size magnitude are encoded into a common GMS representation, and that the congruity effect occurs due to a conflict or a match at this encoding stage (e.g., Arend & Henik, 2015; Cohen Kadosh et al., 2008; Reike & Schwarz, 2017; Schwarz & Heinze, 1998; Szucs & Soltesz, 2008). In addition, because in this paradigm the numerical magnitude is presented symbolically whereas the size magnitude is perceptual, the observed congruity effect also supports the claim that number symbols make use of at least partially shared representations not only with perceptual numerical magnitude, but also perceptual magnitude in other dimensions.

#### Size congruity effect with number words

So far, we discussed the size congruity effect in case of Arabic digits as that is the number representation format with which this effect has been classically and most commonly reported. In the present study, we want to compare congruity effects observed with numerical magnitude and congruity effects observed with scalar adjectives. Having this goal in mind, Arabic digits are not suitable as stimuli since they differ from scalar adjectives not only in their meaning, but also in the fact that digits are presented as one symbol whereas scalar adjectives need to be processed as words before their meaning is accessed. In contrast, number words (i.e., “three,” “five,” etc.) are more like adjectives—they also need to be processed as words before the numerical magnitude is accessed. As discussed below, whether size congruity effect can be observed with number words still remains an open question, so the present study will add evidence on that question as well.

Most classical models of numerical processing assume that there exists a single representation of analog magnitude codes that can be used for numerical magnitude comparison from symbolic input of various notations; these same magnitude codes would be accessed if the stimuli are presented as, e.g., Arabic digits, written number words, spoken number words, etc. (Cipolotti & Butterworth, 1995; Dehaene, 1992; Koechlin et al., 1999; McCloskey, 1992). Nonetheless, empirical evidence shows that there are certain differences in processing different notations that could be attributed to, for example, differences in the amount of experience with a particular notation (Campbell & Epp, 2004), varying processing speed (Cohen Kadosh et al., 2008), or other factors (see Cohen Kadosh et al., 2008 for a discussion).

In a numerical comparison task (with size magnitude as the task-irrelevant dimension), a size congruity effect with number words has been reported in English (Foltz et al., 1984), Hebrew (Cohen Kadosh et al., 2008), and with Japanese Kana numbers (syllabic script close to alphabetic script in English, Ito & Hatta, 2003). In contrast, in a physical size comparison task (with numerical magnitude as the task-irrelevant dimension) the results so far are mixed—the congruity effect has not been observed for Japanese Kana numbers (Ito & Hatta, 2003) but has been reported in Hebrew under some conditions (Cohen Kadosh et al., 2008).

An important aspect that has not been fully taken into account in the previous studies with number words is that the size congruity effect has been shown to be modulated (and masked) by discriminability as well as by variability of the presented stimuli (Algom et al., 1996; Pansky & Algom, 1999). *Discriminability* refers to the psychological difference separating two stimulus values along a dimension, measured in terms of the speed needed to discriminate the two stimuli along this dimension. The second relevant aspect, *variability* refers to the number of different levels of magnitude in each dimension, or how finely grained each dimension is. Both discriminability and variability are thought to influence the salience of each dimension, or the amount of attention that is given to it—the more variable and more discriminable dimension will take more attentional resources. If the irrelevant dimension is more discriminable and variable than the relevant dimension, it will interfere with the relevant dimension simply because it attracted more attentional resources. If the relevant dimension is the more discriminable and variable, the irrelevant dimension will not have an opportunity to interfere because it will not be able to attract enough attentional resources. In their studies, Algom and Pansky demonstrate that only in case discriminability and variability are matched can we conclude that the congruity effect was or was not present specifically due to interference of magnitude codes in each dimension (Algom et al., 1996; Pansky & Algom, 1999). For example, in the study of Ito and Hatta (2003) participants were notably slower in the numerical magnitude comparison task than in the physical size comparison task (the difference was around 250–300 ms), meaning that discriminability was worse for the numerical magnitude than for the physical size in their stimuli. It is then not surprising that they observed a congruity effect when the numerical magnitude was the task-relevant dimension but not when it was the task-irrelevant dimension.^2^

The second study that investigated the size congruity effect with the physical size comparison task, by Cohen Kadosh and colleagues (2008), reported the congruity effect both when the numerical magnitude was task-irrelevant and when the size magnitude was task-irrelevant in one of the experiments. In the critical experiment of this study (Experiment 4), the stimuli in the two dimensions were matched in terms of variability, but still were not matched in terms of discriminability. In fact, the physical size judgements were faster than the numerical magnitude judgements by around 100–300 ms. Whereas they *do* observe a congruity effect despite this mismatch in discriminability of the two dimensions, the pattern of the effects they observed was somewhat different from that observed for Arabic digits within the same study. Specifically, both congruent and incongruent conditions with number words were in fact slower than a third, neutral condition where the numerical dimension (which was task-irrelevant) did not vary between two stimuli (i.e., same number word presented twice on the screen). In contrast, in the parallel experiment with Arabic digits the neutral condition Reaction Times (RT) was between the RTs of the congruent and incongruent condition. In addition, their experiment additionally included a numerical distance manipulation for which they observe RT effects in case of digits, but not in case of number words.

Given that in their studies Arabic digits did interfere with size magnitude processing, whereas number words did not interfere with it or did so with a different pattern of effects, Ito and Hatta (2003) as well as Cohen Kadosh and colleagues (2008) propose that Arabic digits and number words differ in their relation to GMS. Either the number words do not have a strong automatic connection to the GMS representations in this task, or processing number words, unlike Arabic digits, does not recruit GMS representations in general. However, given that neither of these studies fully matched variability and discriminability of the stimuli, more data are needed to draw convincing conclusions regarding shared representations of size magnitude and numerical magnitude for number words.

### Alternative accounts of the source of the size congruity effect

While the size congruity effect has traditionally been seen as evidence for shared representations underlying numerical and size magnitude (e.g., Cohen Kadosh et al., 2008; Schwarz & Heinze, 1998), several alternative accounts of the observed effects have been brought up. In order to conclude that the representations are shared between the two dimensions in our own set of experiments, we have to address these alternative explanations. Two alternative accounts (based on verbal label assignment to each stimulus and attentional capture) that we consider less problematic are described in the *online Supplementary Material B*.

The most important and relevant alternative account suggests that the size congruity effect originates in the decision (i.e., response selection) stage of processing (Faulkenberry et al., 2016; Santens & Verguts, 2011 see also Proctor & Cho, 2006 for another account with similar reasoning). This account is based on the simple fact that in the congruent condition both the task-relevant and the task-irrelevant dimensions (size and numerical magnitude) converge on the same (potential) (motor) response (e.g., *right larger* or *left larger*), whereas in the incongruent condition the relevant and irrelevant dimensions diverge on different (motor) responses. One can imagine that processing of numerical magnitude and size magnitude happens in parallel, using different representations, but both result in a potential motor response option. These motor responses then compete for selection. Importantly, a computational implementation of this account (Verguts et al., 2005) also gives an explanation for the previously mentioned modulation of the congruity effect by the difference between magnitudes in the task-irrelevant dimension. According to this model, the amount of activation passed on to the units deciding between alternative motor responses (decision units) depends on the difference between magnitude values from which the system was choosing. When the difference between them is large, there will be a stronger activation passed on to the potential motor response and this activation will thus have a stronger influence on the decision unit. As a result, when the difference in the task-irrelevant dimension is large, there will be a stronger activation of the response induced by this dimension on the decision units than when the difference on the task-irrelevant dimension is small. Thus, the larger difference on the task-irrelevant dimension will have a stronger impact on the decision units, delaying the decision for the eventual response in the task relevant dimension, and causing a larger congruity effect (see Verguts et al., 2005, for details).

There are several counter-arguments against an account that is exclusively based on the conflict at the decision stage of processing (henceforth, referred to as “decision stage conflict”). First, such an account of the congruity effect (as presented by Santens & Verguts, 2011) predicts that it should arise to an equal extent with different decision polarities (i.e., “choose smaller” task or “choose larger” task) and with different task-relevant and task-irrelevant dimensions, as long as in each case there are two response options compatible with both task-relevant and task-irrelevant dimensions. However, the size congruity effect seems to be modulated by the decision polarity (“choose larger” or “choose smaller”) and differs depending on which dimension is task-relevant (i.e., numerical comparison or physical size comparison task; Arend & Henik, 2015; Tzelgov et al., 1992 see Arend & Henik, 2015 for this argument and supporting evidence). Moreover, ERP studies on the size congruity effect found that a neural correlate of interference is observable both at an early stage of processing (150–250 ms after stimulus presentation), the point when the stimuli are thought to be mapped to magnitude representations, and later stage of processing (300–430 ms), the point when the response is thought to be selected (Szucs & Soltesz, 2008; see also Cohen Kadosh et al., 2007; Schwarz & Heinze, 1998 for converging evidence). While it is difficult to pinpoint the source of an ERP effect, these findings provide evidence that at least part of the congruity effect arises from a conflict at an early processing stage, possibly at level of magnitude representations.

Note that it is also possible that the size congruity effect arises partially due to a conflict at the decision stage of processing and partially due to a conflict at shared representations of size magnitude and numerical magnitude (this has also been suggested by proponents of the response selection account—e.g., Faulkenberry et al., 2016; Santens & Verguts, 2011). In the present study, we collect additional data with the same stimuli but a completely different task to be able to test whether the observed congruity effect originates exclusively from the conflict at the decision stage of processing.

### Present study

In the present series of experiments, in a first step we use the size congruity paradigm to look at the congruity effect between numerical magnitude conveyed by number words and the physical (font) size magnitude of these number words. One group of participants performed a numerical magnitude comparison (Experiment 1a); another group of participants performed a physical size comparison task (i.e., font size comparison; Experiment 1b) on the same stimuli. As discussed above, the existing studies investigating the size congruity effect with number words had unbalanced stimuli in terms of variability and discriminability of magnitudes in the task-relevant and task-irrelevant dimensions. In the present experiments, we balanced variability and discriminability of the stimuli, making it a stronger test case for potential congruity effects than existing studies with number words. We collected data with number words (and not digits) to be able to compare the observed effects with those for scalar adjectives that we are primarily interested in in the present study.

In the next step, we use the reasoning and the experimental set-up of the size congruity paradigm to look at a potential representational overlap between the meaning of scalar adjectives and magnitude representations in GMS. We did so by inspecting the potential interference between the retrieval of the (meaning of) scalar adjectives and presented physical size magnitude. These experiments were parallel to the ones with number words. One group of participants performed a comparison of pairs of scalar adjectives (e.g., “kort-lang” [“short-long”], “laag-hoog” [“low-high”], “licht-zwaar” [“light-heavy”]) in terms of their meaning (Experiment 2a). Specifically, they were asked to judge which of two antonymous adjectives “means more/less of something” while the match with the task-irrelevant font size of these adjectives was manipulated. Henceforth, we refer to the scalar adjective comparison (Experiment 2a) and numerical magnitude comparison (Experiment 1a) as *semantic comparison* tasks. Another group of participants performed a physical size comparison with pairs of scalar adjectives as stimuli (Experiment 2b). Again, the match with the meaning of scalar adjectives was manipulated to create congruent and incongruent trials. Collecting data for number words and scalar adjectives in experiments with parallel designs allows us to compare these two symbolic references to magnitudes. If scalar adjectives and number words make use of GMS representations in the same way, we expect to see parallel congruity effects for both. Alternatively, they may differ either in automaticity or in the source of congruity effect.

To anticipate, we find a reliable congruity effect in case of the semantic comparison tasks with both number words and scalar adjectives (i.e., with the size magnitude being the task-irrelevant dimension), though not in case of physical size comparison tasks. In order to locate the source of this congruity effect (representational overlap vs. decision stage conflict), we followed up these experiments with two additional experiments (Experiment 1c for number words and Experiment 2c for scalar adjectives). These experiments used a different task which asked participants to indicate whether the two presented number words or scalar adjectives were same (e.g., “one-one”) or different (e.g., “one-six”), i.e., they performed a “same”/“different” judgement. The stimuli in the “different” trials (i.e., trials with two different number words or scalar adjectives) were the same pairs as the ones used in the comparison experiments (Experiments 1a, 1b, 2a, 2b). These were the trials of interest that we analysed.

For the same/different task, the shared representations and the decision stage conflict accounts make different predictions for the critical “different” trials. Given that in the “different” trials two different number words (or adjectives) along with two different physical sizes were presented in both congruent and incongruent trials (i.e., trials considered “congruent” and “incongruent” in the comparison tasks), both dimensions should activate the “different” response in congruent and incongruent trials. So no conflict should arise between potential responses from the two dimensions in either type of the trials. Thus, the decision stage conflict account predicts that no congruity effect should be observed in the same/different task. In contrast, because the shared representations account claims that the congruity effect arises from the magnitude code mapping stage of the processing, it still predicts a congruity effect in this task—processing mismatching numerical magnitude and size magnitude should result in a conflict at the level of representations regardless of the exact goal (i.e., the specific response) for which the participant is computing and using these representations. Thus, according to the shared representations account we should still observe faster reaction times in trials congruent than in incongruent trials.

An overview of materials, tasks and goals in each presented experiment is provided in Table 1.

## Experiments 1a and 1b: comparison tasks with number words

Participants saw pairs of number words on the screen and were asked to decide which is numerically larger/smaller (Experiment 1a) or which is presented in larger/smaller font size (Experiment 1b). These experiments follow the classical size congruity paradigm (Besner & Coltheart, 1979; Henik & Tzelgov, 1982) except that number words were presented instead of digits. The same stimuli were presented in both experiments. The number word with a larger numerical magnitude could be presented in a large font size, creating a *congruent* condition, or in a small font size, creating an *incongruent* condition (and correspondingly with the smaller numerical magnitude). We expected to observe a congruity effect—shorter reaction times in the congruent condition than in the incongruent condition. Such a congruity effect would suggest that the magnitude of the task-irrelevant dimension was automatically processed and that it interfered with processing of the magnitude of the task-relevant dimension.

The participants either indicated which of the two items of a trial is larger or they indicated which is smaller in the task-relevant dimension, a manipulation that we will refer to as *decision polarity*. The decision polarity was reversed for every participant in the middle of the experiment.

### Method

#### Participants

These and all other experiments reported in this manuscript were conducted online, in web browsers of the participants. Participants were recruited via Prolific.ac (Palan & Schitter, 2018). All our experiments were in Dutch. To take part in the experiments, participants had to be 18–35 years old, native speakers of Dutch, and born in and currently living in the Netherlands. Each participant was told that the study will take approximately 20 min and was reimbursed for their time with 3.50 British pounds. Participants of all experiments reported in this manuscript were informed about the goal of the study, which data was collected and gave their consent to participate by explicitly clicking on the text “I agree to take part in this experiment.” After data collection, the following participant exclusion criteria were applied: a participant gave incorrect responses in more than 15% of trials, the time spent reading the first instructions of the experiment was less than 10 s, the time spent on the whole experiment was longer than 30 min (measured from when they started the first practice trial). These criteria were applied to ensure that the participants included in the analysis definitely understood the instructions and did not devote time to another task (e.g., opening another website) during the experiment. For each experiment, data collection continued until we reached the desired number of participants meeting the inclusion criteria.

We aimed to collect data from the same number of participants across the experiments with number words and scalar adjectives. The size congruity effect is typically robust and detectable with relatively few participants: previous studies report significant effects with 10–20 participants (e.g., Cohen Kadosh et al., 2008; Henik & Tzelgov, 1982; Kaufmann et al., 2005; Santens & Verguts, 2011). We do not know if the effect size in case of the scalar adjectives will be comparable to the one for the numerical magnitudes, it may in fact be smaller than for numerical magnitude.

Given these considerations, we decided to collect data from 50 participants in each of the experiments.

Fifty-five participants completed Experiment 1a, i.e., the semantic comparison task with number words. Four participants were excluded from the analysis because they read the first instructions in less than 10 s. One further participant was excluded because they spent more than 30 min on the experiment. The mean age of the included participants was 25 years (*SD* = 4.6; 31 male and 19 female). On average, they took approximately 14:40 min to complete the experiment (*SD* = 02:22, min. 12, max. 26).

Fifty-eight participants completed Experiment 1b, i.e., the physical size comparison task with number words. Five participants were excluded from the analysis because they gave incorrect responses in more than 15% of trials. Two participants were excluded because they spent more than 30 min on the experiment. Finally, one participant was excluded because they read the first instructions in less than 10 s. The mean age of the included participants was 25 years (*SD* = 4.6; 32 male and 18 female). On average, they took approximately 14:50 min to complete the experiment (*SD* = 02:37, min. 12, max. 23).

#### Stimuli

Exactly the same stimuli were used across the experiments on semantic comparison (1a) and physical size comparison (1b) with the only difference between the experiments being in the instructions participants received (see *Procedure* below for details).

We used five pairs of number words: “een-zes” [‘one-six’], “twee-acht” [‘two-eight’], “twee-vijf” [‘two-five’], “drie-acht” [‘three-eight’], and “vier-acht” [‘four-eight’], presented in five combinations of font sizes respectively: 41–47 pt, 37–42 pt, 41–46 pt, 38–42 pt, 43–48 pt. Each number word pair was matched with a unique font size pair in order to ensure equal variability in both dimensions. In other words, for example, in case of the pair “een-zes,” ‘een’ was presented in font size 41 pt and “zes” was presented in font size 47 pt in the congruent condition and vice versa in the incongruent condition. Both number words within a pair had the same number of letters in order to avoid a potential confound with the visual difference in the length of words. Note that due to differences in the screen sizes and resolutions of participants’ computers we are only able to provide information about the point sizes of the stimuli, not the real sizes of the stimuli on their screens.

Similarly to previous studies (e.g., Algom et al., 1996; Pansky & Algom, 1999; Santens & Verguts, 2011), comparable discriminability in the task-relevant and task-irrelevant dimensions was achieved by matching the mean reaction time observed for comparison of the number words when both number words of a given pair were presented in the same font size (in our case, this was 44 pt) and for comparison of the font sizes in a meaningless context (in our case, strings of consonants were presented in different font sizes). We collected data in a norming study prior to the experiments from 30 participants recruited from the same population (none of these participants subsequently took part in the actual experiments). For details on this norming study and full results, see the Open Science Framework website: https://osf.io/kh6eb/. The mean RTs and error rates for the selected number word and font size combinations are provided in the *online Supplementary Material* C (Table 1).

The five stimulus pairs of interest^3^ were intermixed with three filler stimulus pairs in order to reduce the possibility that participants will learn responses to specific pairs. These filler pairs were “twee-drie” [‘two-three’], “zeven-negen” [‘seven-nine’], and “drie-vier” [‘three-four’] presented in font sizes 42–46 pt, 38–43 pt, 38–44 pt respectively. In case of filler trials, the discriminability was not matched.

Each of the number word pairs was presented in the congruent (numerically larger number word presented in larger font size) and the incongruent (numerically larger number word presented in smaller font size) condition an equal number of times.

**Figure 1.**
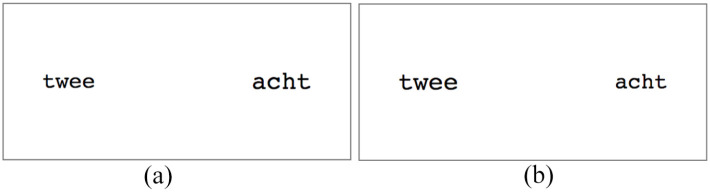
Examples of displays in congruent and incongruent conditions in Experiments 1a and 1b. Font sizes in these examples are 37 pt and 42 pt. (a) congruent condition and (b) incongruent condition.

Examples of displays in the congruent and incongruent conditions are provided in Figure 1.

Each number word in a pair appeared on both sides of the screen in each condition. Each configuration (of congruity and location on the screen) was repeated five times. Finally, participants performed a “choose larger” as well as “choose smaller” tasks (decision polarities). In total, thus, participants saw 8 (number word pairs; 5 pairs of interest and 3 filler pairs) * 2 (levels of congruity) * 2 (sides of the screen) * 5 (repetitions) * 2 (decision polarities) = 320 trials. Out of these trials, 200 were trials of interest and 120 were filler trials. Out of trials of interest, 100 trials were in the “choose larger” decision polarity and the other 100 in the “choose smaller” decision polarity. Within each of the decision polarities, participants saw 50 trials of interest in the congruent condition and 50 trials of interest in the incongruent condition. In each experiment, half of the participants performed the “choose larger” task first (160 trials, after which they were instructed to make decisions with the other decision polarity) and half of the participants performed the “choose smaller” task first.

#### Procedure

The data for all experiments reported in this manuscript have been collected remotely—participants completed the experiments from their own computers in a web browser. Previous studies testing the difference between reaction times observed from an experiment running in a web browser and using traditional lab tools (such as Matlab Psychophysics Toolbox) showed that although there was a time-lag in the reaction times observed in a study running in a web browser (of about 25 ms), there was no difference in terms of the distributions of the RTs and no difference in sensitivity to RT-differences between experimental conditions (de Leeuw & Motz, 2016; Reimers & Stewart, 2015). When it comes to within-participant designs, potential effects should be detected with the same reliability as with traditional lab tools because the equipment stays the same throughout the experiment. A number of classical effects in cognitive psychology have been successfully replicated with data collected online (e.g., Crump et al., 2013; Semmelmann & Weigelt, 2017; Zwaan et al., 2018), leading to the conclusion that online data collection is a suitable and reliable option for hypothesis testing. Finally and most importantly, a recent study which used specifically the size congruity paradigm in web browsers observed data quality comparable to the physical lab-based studies and successfully replicated the classical congruity effects (Kochari, 2019).

The experiments were administered using jsPsych, a JavaScript library for running behavioural experiments in a web browser (https://www.jspsych.org/; de Leeuw, 2015).

In Experiment 1a, participants were instructed to indicate the side of the screen with a larger or smaller number (i.e., numerical magnitude) by pressing a corresponding key on their keyboard. They were told to ignore any other properties of the display. An example was given to demonstrate that it is indeed the numerical magnitude that they should pay attention to. In Experiment 1b, participants were instructed to indicate the side of the screen with a word in larger or smaller font size. In this case too, there was an example showing that they should ignore the numerical magnitude and only pay attention to the font size. Participants were asked to keep their index fingers on two response keys “P” and “Q” and encouraged to respond as soon as possible.

Participants opened the page with the experiment by clicking on a link on the Prolific.ac website. They first read the consent form information and agreed to participate. They were then presented with instructions for the first decision polarity. At this point, the participants were not informed that they will later be asked to make a decision with the reversed polarity. After reading the first instructions, they had a chance to practice the experimental task in four practice trials with stimuli which did not appear in the actual experiments. During the practice trials, they received feedback on whether the given response was correct. The experimental trials of the first decision polarity then followed. There was no feedback given at this stage. The experimental trials were presented in a random order without restrictions, divided into two blocks. There was a break between the blocks. Next, the participants were informed that in the second half of the experiment they will be performing a judgement with the opposite polarity, using the same keyboard keys. They again had a chance to practice, this time on seven practice trials. In the second half of the experiment they again saw trials in a random order without restrictions, divided into two blocks.

Each experimental trial started with a fixation cross in the centre of the screen displayed for 200 ms. It was then replaced by the two stimuli displayed to the left and the right of the middle of the screen for 2000 ms or until the participant pressed a response button. The response was given by pressing either “P” on the keyboard if the stimulus on the right side was the intended response or “Q” if the stimulus on the left side was the intended response. If no response was given within 2000 ms, the trial ended automatically. The interval between the response and onset of the fixation cross of the next trial was 200 ms. In order to reduce effects of anticipating the upcoming stimulus (e.g., Clementz et al., 2002), the interval between the display of the fixation cross and the display of the two trial stimuli was varied randomly between trials—each time it was a random number between 300 and 700 ms. For the same reason, we also added filler trials that were empty (the fixation cross was followed by a blank screen for 500 ms and no response was required) to the experiment. We added 12% of empty filler trials to each experiment, and participants were informed about the presence of such trials in the instructions.

All stimuli were presented in *Courier* monospace font. The distance between the point where the word on the left ended and the centre of the screen was equal to the distance between the centre of the screen and the point where the word on the right started. This distance was same for all trials.

#### Analysis

Only trials in which a correct response was given were included in the analysis of the reaction times. In addition, we excluded all trials in which the RT was shorter than 200 ms as those were likely accidental button presses. Finally, we also excluded all trials in which the RT was longer than the mean RT plus three standard deviations in a given decision polarity for a given participant.

The analysis described here was also used for all other experiments in the present study, so we describe it in full detail. Data were analysed in the R environment (R Core Team, 2020)^4^ and inferences were made by fitting linear mixed effect models using functions in the package *lme4* (Bates et al., 2015). The linear mixed model (LME) models always included fixed effects of the factors *congruity* (congruent vs. incongruent), *decision polarity* (“choose larger” vs. “choose smaller”) and their interaction. Initially, we fitted a model with a maximal random effect structure (Barr et al., 2013), i.e., allowing for by-participant and by-item (i.e., number word pair or adjective pair in further experiments) intercepts as well as varying slopes for each effect. Whenever the maximal model did not converge or resulted in a singular fit, we gradually simplified the random effect structure of the original maximal model by excluding the random effect that accounted for least variance until a non-singular converging model was reached (following one of the recommendations of Barr et al., 2013). The reported *p*-value for each factor was obtained using the Satterthwaite approximation for denominator degrees of freedom as implemented in the R package *lmerTest* (Kuznetsova et al., 2017).

In addition to the frequentist LME models, we also fit parallel Bayesian multilevel models using the package *brms* (Bürkner, 2017, 2018) These models allowed us to quantify how much our data supports the null or the alternative hypothesis (see Nalborczyk et al., 2019; Nicenboim & Vasishth, 2016; Vasishth et al., 2018) for descriptions of Bayesian multilevel models in the context of psycholinguistic research]. We chose an ex-gaussian distribution model because it provides a considerably better fit for reaction time data which is typically (and also clearly in the present studies) right-skewed (Lindelø v, 2020; Rousselet & Wilcox, 2019). In addition, examination of posterior predictive values generated by models with a gaussian distribution and an ex-gaussian distribution showed that the latter model was overwhelmingly better able to predict values close to the data we observed. The random effects structure was maximal as described above. We used a normally distributed prior with mean 0 and standard deviation 100 ms for the population-level (i.e., fixed) effects. Such a prior meant that we were 95% certain that the effect of congruity, task and interaction was between -200 and 200 ms. We ran additional models with population-level effect prior SDs 200 and 400. Because the estimates resulting with these priors were extremely close to those with *SD* = 100, we do not report them here. Full results of these models are available in the Open Science Framework website: https://osf.io/kh6eb/.

The priors for the remaining parameters were left as default. The models were fit with four chains and 5000 iterations half of which were the warm-up phase. Model convergence was verified by making sure that there were no divergent transitions, Rhat values were close to one, and by examining the trace plots.^5^ We inspected mean estimates for the effects of interest along with 95% credible intervals (CrI) of the posterior estimate. The 95% CrI should be interpreted as containing the true value of the effect with 95% probability. To quantify the evidence provided by the data for or against the effects of interest being zero, we calculated Bayes Factor values using Savage–Dickey density ratio method (Dickey & Lientz, 1970; Wagenmakers et al., 2010). Our null hypothesis here was that the effect is exactly zero, whereas the alternative hypothesis was that the effect is not exactly zero (note that this is a two-sided test). This calculation gave us a BF_01_ (how much the collected data increases our confidence that the effect is exactly zero relative to how confident we were about it before the data was collected, i.e., relative to the prior described above) or vice versa, BF_10_ (how much the collected data increases our confidence that that the effect is *not* exactly zero relative to how confident we were about it before the data was collected). Note that we report BF_01_ or BF_10_ depending on which one was larger. We interpreted BFs below 3 as inconclusive, above 3 as moderate evidence, and BFs above 10 as strong evidence in favour of one hypothesis over another (Jeffreys, 1998).

Raw data, analysis scripts and full model results for all experiments presented in this manuscript are provided in the online materials available on Open Science Framework under https://osf.io/kh6eb/.

### Results

In Experiment 1a, i.e., the semantic comparison task with number words, participants included in the analysis made 3.58% errors in the whole experiment on average (min. 0%, max 10%). Data cleaning procedure resulted in exclusion of RTs of 2.92% of trials of interest (excluded incorrect responses are also counted here). The resulting mean RTs and error rates per congruity overall and in each decision polarity are given in Table 2. Mean RTs and error rates per number word pair across the decision polarities are given in Table 3.

**Table 2.** Mean RT (*SD*), error rate overall and for each decision polarity in Experiment 1a, semantic comparison with number words.

Decision polarity	Congruent	Incongruent
Overall	720 (182) ms, 1.88%	750 (184) ms, 2.16%
‘Choose larger’	699 (166) ms, 1.96%	729 (172) ms, 2.56%
‘Choose smaller’	742 (195) ms, 1.80%	772 (194) ms, 1.76%

**Table 3.** Mean RT (*SD*), error rate per number word pair (both decision polarities) in Experiment 1a, semantic comparison with number words.

Number word pair	Congruent	Incongruent
‘een-zes’	646 (137) ms, 0.2%	672 (152) ms, 0.3%
‘twee-acht’	697 (176) ms, 0.7%	734 (166) ms, 1.3%
‘twee-vijf’	708 (168) ms, 0.8%	735 (174) ms, 0.7%
‘drie-acht’	768 (193) ms, 2.3%	799 (189) ms, 3.8%
‘vier-acht’	788 (197) ms, 5.4%	817 (203) ms, 4.7%

The linear mixed effect model with maximal random effect structure for Experiment 1a data resulted in a singular fit. The random effect structure was gradually simplified to achieve a converging non-singular model fit. The final model included varying intercepts per-participant and per-item (i.e., per number word pair) as well as varying slopes for the effect of decision polarity in both cases. There was a significant main effect of congruity (β = 29, *SE* = 4.0, *t* = 7.37, *p* < .0001) and a significant main effect of decision polarity (β = 43, *SE* = 16.7, *t* = 2.6, *p* = .038). The interaction effect was not significant (β = −1, *SE* = 5.7, *t* = −0.22, *p* = .82). For this and all further analyses, the result of the maximal random effect structure model (resulting in a singular fit) did not contradict the results of the model with the simplified random effect structure; results of all models can be inspected in the Open Science Framework website: https://osf.io/kh6eb/.

The Bayesian LME model estimated for the main effect of congruity $\hat{\beta }$ = 25 ms, 95% CrI = [15.93 35.64], BF_10_ = 14.3; for the main effect of decision polarity $\hat{\beta }$ = 27 ms, 95% CrI = [-14.95 69.09], BF_01_ = 2.9, for the interaction between congruity and decision polarity $\hat{\beta }$ = 3 ms, 95% CrI = [-11.52 17.93], BF_01_ = 29.2. Thus, there was strong evidence that the congruity effect was not zero, no clear evidence for or against the decision polarity effect being zero (though most of the weight of the posterior distribution is on one side of zero—in favour of the alternative hypothesis) and strong evidence that the interaction between congruity and decision polarity was zero.

In Experiment 1b, i.e., the physical size comparison task with number words, participants included in the analysis made 5.78% errors in the whole experiment on average (min. 2%, max. 12%). Data cleaning procedure resulted in exclusion of RTs of 8.36% of trials of interest (excluded incorrect responses are also counted here). The resulting mean RTs and error rates per congruity overall and in each decision polarity are given in Table 4. Mean RTs and error rates per number word pair across the decision polarities are given in Table 5.

**Table 4.** Mean RT (*SD*), error rate overall and for each decision polarity in Experiment 1b, physical size comparison with number words.

Decision polarity	Congruent	Incongruent
Overall	744 (241) ms, 5.06%	788 (259) ms, 8.62%
‘Choose larger’	733 (239) ms, 5.12%	775 (259) ms, 7.64%
‘Choose smaller’	757 (243) ms, 5.00%	803 (260) ms, 9.60%

**Table 5.** Mean RT (*SD*), error rate per number word pair (both decision polarities) in Experiment 1b, physical size comparison with number words.

Number word pair	Congruent	Incongruent
‘een-zes’	690 (198) ms, 1.60%	797 (246) ms, 7.51%
‘twee-acht’	721 (211) ms, 2.10%	748 (235) ms, 2.80%
‘twee-vijf’	663 (186) ms, 1.10%	936 (314) ms, 26.60%
‘drie-acht’	853 (290) ms, 15.2%	751 (238) ms, 2.70%
‘vier-acht’	821 (262) ms, 5.30%	756 (235) ms, 3.50%

The LME model with maximal random effect structure for Experiment 1b also resulted in a singular fit. The random effect structure was gradually simplified to achieve a converging non-singular model fit. The final model included a per-participant intercept allowing for varying slopes for the effect of decision polarity and allowed for varying random slopes for the congruity effect by-item. None of the effects were significant (main effect of congruity - β = 44, *SE* = 67.4, *t* = 0.65, *p* = .54; main effect of decision polarity - β = 22, *SE* = 15.7, *t* = 1.44, *p* = .15; interaction of congruity and decision polarity - β = 7, *SE* = 8.4, *t* = 0.86, *p* = .38). Note that whereas in the overall means there does seem to be a difference in RTs between congruent and incongruent conditions, closer inspection of the RTs observed for each of the number word pairs (as can be seen in Table 5) shows that in case of two number word pairs the RTs were in fact shorter for the incongruent than for the congruent condition. This is reflected in the non-significant congruity effect in the model.

The Bayesian LME model estimated for the main effect of congruity $\hat{\beta }$ = 20 ms, 95% CrI = [-57.28 94.46], BF_01_ = 2.2; for the main effect of decision polarity $\hat{\beta }$ = 10 ms, 95% CrI = [-36.26 53.72], BF_01_ = 4.32, for the interaction between congruity and decision polarity $\hat{\beta }$ = 6 ms, 95% CrI = [-31.23 42.98], BF_01_ = 6.06. Thus, there is no clear evidence for or against the congruity effect being zero, moderate evidence that decision polarity effect is zero and moderate evidence that the interaction between congruity and decision polarity is zero.

### Interim discussion

Let us first consider the implications of the results of the numerical magnitude comparison task (Experiment 1a). In this task, we observed a clear congruity effect that was stable across different number word pairs. Observing the congruity effect here is consistent with previous studies that administered the size congruity paradigm with number words (Cohen Kadosh et al., 2008; Ito & Hatta, 2003). Importantly, unlike the previous studies, we have matched the stimuli in the task-relevant and task-irrelevant dimensions in terms of both variability and discriminability. In addition, we collected our data in a language for which the size congruity effect with number words has not previously been reported—Dutch. Thus, these results support the robustness of the size congruity effect in the numerical comparison task with number words.

The reaction times were descriptively shorter in trials where the participants were asked to choose a numerically larger number word than in the trials where the participants were asked to choose a numerically smaller number word; this effect was significant in the frequentist LME but inconclusive in the Bayesian LME estimates. In general, the shorter RTs for the “choose larger” decision polarity is consistent with the pattern previously reported for Arabic digits (Arend & Henik, 2015). Importantly, we observed a congruity effect for both decision polarities. This is consistent with previous studies that administered both decision polarities in the size congruity task with Arabic digits (Arend & Henik, 2015; Tzelgov et al., 1992). To our knowledge, no previous studies administered different decision polarities with number words, so this is a first demonstration of the congruity effect with the “choose smaller” decision polarity.

However, unlike in the previous studies with Arabic digits (Arend & Henik, 2015; Tzelgov et al., 1992), the size of the congruity effect in our experiment was not modulated by the polarity of instructions. In the studies with Arabic digits, a larger congruity effect was reported for the “choose larger” decision polarity than for the “choose smaller” decision polarity. We, on the contrary, have strong evidence that the interaction of congruity and decision polarity is zero in our data.

Let us now turn to the results of the physical size comparison task (Experiment 1b). In this task, the difference between the congruent and incongruent conditions was not consistent across different number word pairs. We have inconclusive evidence for or against the congruity effect being zero (BF_01_ = 2.2) though the null hypothesis is supported by the data slightly more than the alternative hypothesis. The lack of a significant congruity effect in this task is consistent with results of some previous studies (Cohen Kadosh et al., 2008; Ito & Hatta, 2003 Experiment 1). Recall that there was only one study to date reporting a size congruity effect with number words in the physical size comparison task (Cohen Kadosh et al., 2008 Experiment 4). The earlier discussed discriminability mismatch in that study (as opposed to discriminability match in our study) cannot explain the different findings because in that study the size magnitude was easier to discriminate than that of the numerical magnitude which, according to Algom and Pansky (Algom et al., 1996; Pansky & Algom, 1999) predicts that numerical magnitude should not interfere with processing size magnitude. They observed the congruity effect despite the discriminability mismatch.

Interestingly, the fact that we do not observe a significant congruity effect in the physical size comparison task despite using exactly the same stimuli as in the numerical magnitude comparison task goes against the prediction of the decision stage conflict account (Santens & Verguts, 2011). Recall that according to that account, the congruity effect should be observed regardless of which exact dimension is task-relevant, as long as the decision alternatives in two tasks are exactly the same. It is also problematic for the shared magnitude code representations overlap because according to this account interference should arise whenever magnitudes in two dimensions are retrieved/computed regardless of which one is task-relevant and which one is task-irrelevant. Thus, both of these accounts have to be somehow modified in order to explain the lack of the congruity effect in the physical size comparison task. We investigate the source of the congruity effect in the numerical comparison task before making conclusions.

In order to investigate whether the congruity effect that we observed in the numerical magnitude comparison task originates from the representational overlap at the level of magnitude codes or from a conflict at the decision stage, we conducted a follow-up experiment in which participants were asked to make a same/different judgement on the same stimuli.

## Experiment 1c: same/different task with number words

We observed a significant difference between the congruent and the incongruent condition in the semantic comparison task with number words (Experiment 1a). Under the classical interpretation, this congruity effect arises from the overlapping magnitude code representations for the numerical magnitudes that are evoked by the number words and for the size magnitudes that are evoked by the font size difference. Therefore, this effect is seen as evidence in favour of number words evoking GMS representations. Under the alternative account, the congruity effect arises due to a conflict at the decision stage, simply because both the task-relevant and the task-irrelevant dimensions are processed in parallel. Since the response options are compatible for both of them, they subsequently compete for the response that should be given in case of the incongruent condition (e.g., the numerical magnitude dimension evokes a “right larger” response, whereas the size magnitude dimension evokes a “left larger” response.), but not in case of the congruent condition (e.g., both magnitude dimensions evoke a “right larger” response). Under this interpretation, the congruity effect does not say anything about the interaction of GMS and numerical magnitudes conveyed by number words. It should be noted that while the congruity effect with number words has previously been interpreted as evidence in favour of the representational overlap account (Cohen Kadosh et al., 2008; Ito & Hatta, 2003), none of these previous studies have ruled out the decision stage conflict account. This is what we will look into now.

To tap into the origin of this congruity effect, we constructed a novel experiment where in part of the trials (the critical trials of the present experiment) the participants saw exactly the same stimuli as in the comparison task, but were asked to make a different decision: they had to decide whether the two presented words of a trial were repetitions of the same word or two different words. In trials with the same stimuli as in the comparison task (Experiment 1a) two different number words were presented and thus, participants had to respond “different” (and this held for both the congruent and the incongruent trials of Experiment 1a). We analysed the reaction times for these trials. We added trials where participants saw the same number word on two sides of the screen (e.g., “twee-twee” [‘two-two’] or “acht-acht” [‘eight-eight’]), and on these trials participants were supposed to respond “same.” These trials were not analysed.

The representational overlap account and the decision stage conflict account make different predictions for the “different” trials of the same/different task. These predictions are illustrated in Table 6 below. See the discussion of the same/different task above under *Present study* for the reasoning behind these predictions.

**Table 6.** Predictions for differences between conditions under the representational overlap and decision stage conflict accounts of the size congruity effect for the comparison task with “choose larger” decision polarity and for the same/different task. “Left” and “right” as well as “same” and “different” refer to response alternatives in the task.

	Comparison task		Same/different task	
	Prediction under representational overlap	Prediction under decision stage conflict	Prediction under representational overlap	Prediction under decision stage conflict
Congruent: twee **acht**	Magnitude code match	Font: *right*, number: *right*	Magnitude code match	Font: *different*, number: *different*
Incongruent: **twee acht**	Magnitude code mismatch	Font: *left*, number: *right*	Magnitude code mismatch	Font: *different*, number: *different*

A similar reasoning as the one we are using here in order to disentangle the two potential sources of this congruity effect has previously been applied in a study investigating subliminal priming of area size judgements by numerical magnitude, using Arabic digits (Lourenco et al., 2016, Experiment 2).

### Method

#### Participants

Because we had a restricted set of potential participants meeting the criteria in the pool of registered users of Prolific.ac, participation in this task was open to those who already completed the comparison task with scalar adjectives (Experiment 2a for which the data was collected at an earlier point in time). These participants have not seen number word stimuli before and have not completed a task requiring them to pay attention to the physical size of stimuli, so we did not expect them to be in any way different from completely naive participants. Of the 50 participants included in the analyses of this experiment, 25 participants have previously completed Experiment 2a; the time interval between taking part in the two experiments was at least 7 days.

Fifty-five participants completed the experiment. Three participants were excluded from the analysis because they read the first instructions in less than 10 s. Two further participants were excluded because they spent more than 30 min on the experiment. The mean age of the included participants was 25 years (*SD* = 4.9; 33 male and 17 female). On average, they took approximately 14:43 min to complete the experiment (*SD* = 02:37, min. 12, max. 25).

#### Stimuli

We used the same number word and font size combinations as for Experiments 1a and 1b to construct trials with an expected “different” response. This means that we had five number word and font size combinations of interest as well as three filler combinations. Each number word in a pair appeared on both sides of the screen. In addition, we added trials with an expected “same” response. Here, we presented the same number word on both sides of the screen albeit still in two different font sizes according to the font sizes that this number word was displayed in in the comparison tasks. This was done to keep these “same” trials as similar as possible to the “different” trials. For example, the pair “twee-acht” was presented in font sizes 41 pt and 47 pt in “different” trials (i.e., as in Experiments 1a and 1b). In addition, “twee-twee” and “acht-acht” were presented in font sizes 41 pt and 47 pt to create “same” trials. Each font size appeared on both sides of the screen. The “same” trials were not analysed.

The proportion of “different” and “same” trials was 60:40 rather than balanced 50:50 similarly to the proportion that has been used in previous studies using the same-different paradigm (see Wong & Szücs, 2013, for reasoning for this choice).

Each participant saw 320 trials in total - 120 “different” trials of interest, 80 “same” trials with the same number word pairs as well as 120 filler trials. Each participant saw 60 trials of interest in the congruent condition in terms of magnitude (numerically larger/smaller number word presented in larger/smaller font size) and 60 trials of interest in the incongruent condition in terms of magnitude (numerically larger/smaller number word presented in smaller/larger font size). Because all of these were “different” trials, comparison between these conditions could be made without a potential confound of the given response. The reaction times of the “same” trials were not compared to the “different” trials since participants gave a different response here; we only provide the mean RT for this condition.

#### Procedure

The experimental procedure was identical to that of Experiments 1a and 1b except for instructions, response buttons, and the number of practice stimuli. Participants were instructed to indicate whether they saw the same word on both sides of the screen or different words. Half of the participants were told to press “F” for the “same” response and “J” for the “different” response in the first half of the experiment and vice versa for the second half of the experiment. The other half of participants received this response button mapping in the reversed order. Participants had a chance to practice both response mappings with feedback (at the beginning, when they read the first instructions and in the middle of the experiment, when the response button mapping was reversed). Because remembering the buttons for the same/different judgement might be more demanding than pressing a button on the side of the screen corresponding to a larger/smaller number, we included more practice items—10 items for each response button mapping.

### Results

Participants included in the analysis made 3.42% errors in the whole experiment on average (min. 0%, max 9%). Data cleaning procedure resulted in exclusion of RTs of 4.63% (excluded incorrect responses are also counted here) of “different” trials. The mean reaction time in the congruent condition was 714 ms (*SD* = 175 ms, error rate: 2.7%), in the incongruent condition 719 ms (*SD* = 182 ms, error rate: 2.2%) and in the “same” trials it was 700 ms (*SD* = 159 ms, error rate: 4.4%). Notice that the reaction times were overall somewhat faster for the “same” decision than for the “different” decision. These “same” trials were not analysed, so we now focus on the congruent and incongruent conditions within “different” trials. Mean RTs and error rates per number word pair in each condition are given in Table 7.

**Table 7.** Mean RT (*SD*), error rate per number word pair in “different” trials in Experiment 1c, same/different task with number words.

Number word pair	Congruent	Incongruent
‘een-zes’	690 (159) ms, 2.34%	706 (169) ms, 1.33%
‘twee-acht’	716 (174) ms, 2.50%	734 (185) ms, 2%
‘twee-vijf’	694 (163) ms, 1.84%	703 (169) ms, 1.67%
‘drie-acht’	754 (190) ms, 4.50%	736 (192) ms, 3.17%
‘vier-acht’	720 (185) ms, 2.33%	720 (194) ms, 2.84%

The frequentist LME model with maximal random effect structure included a main effect of congruity and allowed for varying intercepts per-item and per-participant as well as varying slopes for the congruity effect in each case. This model did not converge. Exclusion of the varying slopes for the congruity effect per-participant resulted in a converging fit. The congruity effect was not significant (β = 4, *SE* = 6.5, *t* = 0.62, *p* = .56). The Bayesian LME model estimated for the congruity effect $\hat{\beta }$ = 3 ms, 95% CrI = [-17.04 25.56], BF_01_ = 10.81; thus, there was strong evidence for the congruity effect being zero.

To explore the data further, we looked at whether the participants perhaps learnt to ignore the numerical magnitude of the number words over the course of the experiment. To explore this possibility, we looked at the difference in the mean reaction times between the first half and the second half of the experiment. Indeed, descriptively the difference in the mean RTs was somewhat larger in the first half of the experiment (congruent: 726 ms [*SD* = 181], incongruent: 732 ms [*SD* = 180]) than in the second half (congruent: 702 ms [*SD* = 168], incogruent: 706 ms [*SD* = 183]). Nonetheless, even in the first half of the experiment the congruity effect was not present. In the Bayesian model, there was moderate evidence for the interaction between the experiment half and congruity being zero ( $\hat{\beta }$ = -6 ms, 95% CrI = [-28.3 14.27], BF_01_ = 8.33). The frequentist LME models with a reasonable random effect structure did not converge, so we do not report frequentist LME results here.

### Interim discussion

We reasoned that if the congruity effect observed in the numerical comparison task (Experiment 1a) is explained purely by a conflict at the decision stage of processing, it should disappear when the response alternatives are such that they do not allow for such a conflict to arise. The data obtained with the same/different task indeed show that the congruity effect disappeared. We conclude that the congruity effect observed in the numerical comparison task with number words is likely to be driven by the conflict at the decision stage. Together with the lack of the congruity effect in the physical size comparison task with number words, this means that in the present study we do not observe any evidence for the recruitment of GMS during number word processing. Of course, it is possible that number words do recruit GMS, but that the tasks we used are not adequate for showing an involvement of GMS. But this might then hold also for all previous studies as well. We will therefore discuss these results in the wider context of research on number symbol processing in the *General Discussion* section.

Alternatively, GMS could still have been recruited in the size congruity task but not the same/different task leading to a congruity effect in the former but not in the latter case. This could be because, for example, the size congruity task explicitly required magnitude comparison whereas the same/different task did not. Such a possibility is in accordance with, for example, existence of the asemantic processing route within the number words (and digits) postulated in the triple-code model of number processing (Dehaene, 1992). Specifically, in the same/different task perhaps the number word comparisons were performed from the memory about number words themselves, without triggering activation of numerical representations corresponding to the magnitudes of the number words. Although we cannot exclude this possibility based on our experiments, we prefer the simpler explanation that the observed congruity effect originated at the decision stage of processing. In particular, this interpretation avoids the necessity of postulating different processing modes depending on the task that has to be carried out. Therefore, in the *General discussion* section will focus on the conflict at the decision stage of processing as the most likely mechanism behind the observed effects.

Note that even if number word meaning does not interact with GMS representations, it is still possible that scalar adjectives’ meaning does so given the differences between the properties of number symbols and scalar adjectives. Specifically, as we discussed in the *Introduction*, number symbols may not be compatible with GMS representations because they refer to exact, discrete quantities. In contrast, scalar adjectives do not refer to discrete magnitudes, so they are more compatible with GMS representations than number symbols are.

Even though the results of the present same/different task strongly suggest that the congruity effect in the size congruity paradigm originates at the decision stage, we still used this paradigm to look into processing of the scalar adjectives as well for several reasons. First, as discussed in the *Introduction*, there is evidence of this paradigm tapping into the interaction of magnitude codes of the task-relevant and task-irrelevant dimensions at least in case of Arabic digits and size magnitude. Second and more importantly, given that no previous study has looked at the possible interaction of scalar adjective meaning and GMS representations, comparing behavioural effects with scalar adjectives to those observed with number words in the same paradigm would be a good starting point for this line of research. If we do observe a size congruity effect in case of scalar adjectives, we now know that (given the present results) it is likely to be at least partially driven by the presence of a conflict at the decision stage. It is, however, possible that a congruity effect for scalar adjectives is also partially driven by the representational overlap of scalar adjective meanings and size magnitude. Thus, we will again need to investigate whether the congruity effect originates purely from a conflict at the decision stage in a same/different task.

## Experiments 2a and 2b: comparison tasks with scalar adjectives

In the central experiments of this project, we use the reasoning and the experimental set-up of the size congruity paradigm to look at scalar adjectives and magnitude representations in GMS. In Experiments 2a and 2b, participants saw pairs of scalar adjectives on the screen and were asked to make a decision about their meaning (Experiment 2a) or about the font size in which they were presented (Experiment 2b). The same stimuli were presented in both experiments. The experimental design, procedures and number of trials for these experiments were identical to Experiments 1a and 1b with number words.

In the semantic comparison task with scalar adjectives (Experiment 2a), we employed a novel task. The participants saw pairs of antonymous scalar adjectives referring to continuous property dimensions (e.g.,’kort-lang’ [‘short-long’], “laag-hoog” [‘low-high’], “licht-zwaar” [‘light-heavy’]) in different font size combinations. Note that we did not use the adjective pairs that would be used to describe the physical size contrast itself, i.e., “large-small.” Participants were asked to indicate the adjective in the pair that “means more/less of something,” and they were given several examples such as “young-old” where “old” refers to *more* in terms of age. Thus, for this task participants had to understand the dimension which the adjectives describe, and they had to decide which of the adjectives refers to more/less on this particular dimension. The exact instructions and examples given to the participants are provided in the *Procedure* section below. The low error rate that we observed (between 0.83% and 7%) demonstrates that the participants did not have any difficulty with this task. The physical size comparison task with scalar adjectives (Experiment 2b) was the same as for number words (Experiment 1b)—participants were asked to choose the word that was printed in the larger/smaller font size. They received same instructions as participants in physical size comparison with number words (Experiment 1b).

As for the experiments with number words, we attempted to match the two dimensions (meaning and physical size magnitude) in both variability and discriminability as much as possible. However, despite these attempts, it was not possible to match stimuli in terms of discriminability—the general speed for processing adjective meaning was slower than that for processing font size. This was the case for the following reason. Discriminability of stimuli in the task-relevant and task-irrelevant dimensions is matched by matching the reaction times of the judgements in each of the dimensions separately. The reaction times depend on the difficulty of the comparison in the respective task-relevant dimension. There is no way to manipulate the speed of the symbolic meaning dimension, so this speed is a given fact. By contrast, we can manipulate the physical size judgement—for example, we can make it slower by making the font size difference smaller. However, making the font size differences increasingly smaller also leads to a larger number of errors which is undesirable because we would like to have a roughly comparable error rate in the two dimensions as well. As a result of this, it was not possible to match discriminability fully. As an alternative to full matching, we opted for choosing font size combinations that were closest to the symbolic judgement RTs for the individual specific adjectives pairs.

Let us consider how the difference discriminability in the two dimensions can affect our results. The reaction times for the physical size comparison of font sizes used in the present study was on average 87 ms shorter than the reaction time for the semantic comparison of the adjective pairs (see *Supplementary Material C*, Table 2). According to the findings of Algom and Pansky (Algom et al., 1996; Pansky & Algom, 1999), the fact that the discriminability of physical size is easier than that of scalar adjective pairs means that physical size will attract more attentional resources than the numerical magnitude. For semantic comparison task this means that the congruity effect may arise simply because physical size is more salient and attracts the attentional resources. If this is the case, the congruity effect should be different for each of the stimulus pairs depending on how large the difference in discriminability between the task-relevant and task-irrelevant dimensions is. We consider this possibility in the *Interim discussion* of these experiments below. For the physical size comparison task the discriminability difference means that the adjective meaning may not be able to interfere with the physical size comparison simply because it will not be able to attract enough attentional resources (i.e., it may not be processed fast enough). However, recall that Cohen Kadosh and colleagues (2008) have observed a significant congruity effect in a physical size comparison task with number words despite a larger mismatch in discriminability. Thus, possibly in case of our physical size comparison task we will still be able to observe a congruity effect (especially given that the mismatch in discriminability is in fact smaller than in the study of Cohen Kadosh and colleagues where it was 100–300 ms). Nonetheless, if we do not observe a congruity effect, we will not be able to completely rule out the possibility that discriminability difference did not allow for this effect to emerge.

### Method

#### Participants

Fifty-nine participants completed Experiment 2a, i.e., the semantic comparison task with scalar adjectives. Six participants were excluded from the analysis because they gave incorrect responses in more than 15% of trials. Two participants were excluded because they read the first instructions in less than 10 s. One further participant was excluded because they spent more than 30 min on the experiment. The mean age of the included participants was 25 years (*SD* = 4.9; 33 male and 17 female). On average, they took approximately 15:01 min to complete the experiment (*SD* = 01:55, min. 12, max. 20).

Sixty-three participants completed Experiment 2b, i.e., the physical size comparison task with scalar adjectives. Eleven participants were excluded from the analysis because they gave incorrect responses in more than 15% of trials. One participant was excluded because the data for half of the trials was lost due to technical reasons. Finally, one participant was excluded because they spent more than 30 min on the experiment. The mean age of the included participants was 23 years (*SD* = 4.2; 30 male and 20 female). On average, they took approximately 15:24 min to complete the experiment (*SD* = 02:24, min. 12, max. 24).

#### Stimuli

The configuration of stimuli, number of trials of interest and filler trials was parallel to the ones described for Experiments 1a and 1b with number words. Here and in the rest of the methods section, we only mention the differences from the methods described for number words.

We used five pairs of scalar adjective pairs: “kort-lang” [‘short-long’], “laag-hoog” [‘low-high’], “licht-zwaar” [‘light-heavy’], “dun-dik” [‘thin-thick’], and “stil-luid” [‘quiet-loud’], presented in five combinations of font sizes, respectively: 43–48 pt, 41–47 pt, 37–42 pt, 38–42 pt, 41–46 pt. In order to match the task dimensions on discriminability, we collected data in a norming study prior to the experiments from the same 30 participants that completed the norming for number words (none of these participants took part in the actual experiments; see the Open Science Framework website: https://osf.io/kh6eb/ for details on this norming study). Because the participants were in general considerably slower on judgements of adjective meanings than on font size judgements, it was not possible to fully match the scalar adjective pairs with font size combinations in terms of reaction times. Instead, we chose font size combinations that were closest to the adjectives pairs in terms of RTs. The mean RTs and error rates observed for the selected scalar adjective and font size pairs are provided in *Supplementary Material C* (Table 8).

**Table 8.** Mean RT (*SD*), error rate overall and for each decision polarity in Experiment 2a, semantic comparison with scalar adjectives.

Decision polarity	Congruent	Incongruent
Overall	846 (228) ms, 5.04%	858 (219) ms, 5.48%
‘Choose more’	833 (220) ms, 5.12%	848 (212) ms, 5.24%
‘Choose less’	861 (236) ms, 4.96%	869 (226) ms, 5.72%

The five scalar adjective pairs of interest were intermixed with three further filler pairs: “weinig-veel” [‘few/little-many/much’], “smal-breed” [‘narrow-broad’], and “langzaam-snel” [‘slow-fast’] presented in font sizes 42–46 pt, 38–43 pt, 38–44 pt, respectively. In case of filler trials, the discriminability was not matched.

**Figure 2.**
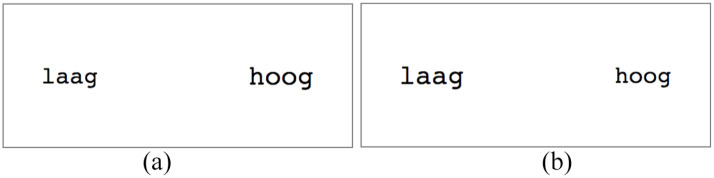
Examples of displays in congruent and incongruent conditions in Experiments 2a and 2b. (a) congruent condition and (b) incongruent condition.

Examples of displays in congruent and incongruent conditions are shown in Figure 2.

#### Procedure

The experimental procedure was identical to that of the Experiments 1a and 1b except for instructions. The following instructions were given to the participants who completed semantic comparison task: “In this experiment, you will see two words at the center of the screen. Your task is to indicate which of the two words means *more of something* by pressing the corresponding key on your keyboard. This means more in terms of what the meaning of the word refers to. For example, in the pair ‘*ol*,’ and ‘*youn*,,’ ‘old’ is more in terms of age. In the pair ‘*expensiv*,’ and ‘*chea*,,’ ‘expensive’ is more in terms of price. In the pair ‘*a lo*,’ and ‘*on*,,’ ‘a lot’ is more in terms of quantity. If the word that means more is on the right side, press ‘P’ and if it is on the left side, press ‘Q.’ For example, you may see ‘old young.’ In this case, the word which means more is ‘old,’ on the left side, and you should press ‘Q.’ If it was ‘young old,’ then you would need to press ‘P.’ You should only compare the meaning of the two words and ignore the other properties. [..].” For the “choose less” decision polarity, the instructions were identical except “more” was substituted by “less” and examples were adjusted. In the practice trials, participants saw the adjective pairs given as examples in the instructions intermixed with other pairs (e.g., “full-empty,” ‘fat-slim,’ etc.). See the Open Science Framework website: https://osf.io/kh6eb/ for full instructions in Dutch as well as all practice trials. None of the adjectives that appeared as an example or in practice trials appeared in the experimental trials.

The texts of the instructions given to the participants who completed physical size comparison with scalar adjectives were identical to those given in the physical size comparison with number words (Experiment 1b). Participants were instructed to indicate the side of the screen with a word in larger or smaller font size. Examples and practice trials given to participants in Experiment 2b were different, however. Here, they saw some scalar adjective pairs (e.g., “old-young,” ‘full-empty’) and some colour adjective pairs (e.g., “red-blue”) as an example and in the practice trials. None of the adjectives that appeared as an example or in a practice trial appeared in the experimental trials.

### Results

In Experiment 2a, i.e., the semantic comparison task with scalar adjectives, participants included in the analysis made 5.16% errors in the whole experiment on average (min. 0%, max 13%). Data cleaning procedure resulted in exclusion of RTs of 6.6% of trials of interest (excluded incorrect responses are also counted here). The resulting mean RTs and error rates per congruity overall and in each decision polarity are given in Table 8. Mean RTs and error rates per adjective pair across decision polarities are given in Table 9.

**Table 9.** Mean RT (*SD*), error rate per adjective pair (both decision polarities) in Experiment 2a, semantic comparison with scalar adjectives.

Adjective pair	Congruent	Incongruent
‘kort-lang’	889 (239) ms, 7.8%	903 (231) ms, 9.7%
‘laag-hoog’	806 (210) ms, 3.8%	825 (202) ms, 4.3%
‘licht-zwaar’	805 (212) ms, 2.0%	815 (201) ms, 2.0%
‘dun-dik’	853 (228) ms, 6.5%	864 (231) ms, 6.5%
‘stil-luid’	887 (238) ms, 5.11%	891 (217) ms, 4.9%

The model with maximal random effect structure for Experiment 2a did not converge. The random effect structure was gradually simplified to achieve a converging non-singular model fit. The final model included a varying intercept per-item as well as varying intercept per-participant allowing for varying slopes for the effect of decision polarity. In this model, the main effect of congruity was significant (β = 16, *SE* = 5.7, *t* = 2.82, *p* = .005) along with the main effect of decision polarity (β = 28, *SE* = 11.9, *t* = 2.35, *p* = .022). The interaction of congruity and decision polarity was not significant (β = −8, *SE* = 8.1, *t* = −1.05, *p* = .28). The Bayesian LME model estimated for the main effect of congruity $\hat{\beta }$ = 19 ms, 95% CrI = [7.71 31.86], BF_10_ = 9.09; for the main effect of decision polarity $\hat{\beta }$ = 24 ms, 95% CrI = [-14.1 59.95], BF_01_ = 1.86, for the interaction between congruity and decision polarity $\hat{\beta }$ = -6 ms, 95% CrI = [-25.85 13.1], BF_01_ = 8.94. Thus, there is moderate evidence that the congruity effect is not zero, no clear evidence for or against the decision polarity effect being zero and moderate evidence that the interaction between congruity and decision polarity is zero.

In Experiment 2b, i.e., the physical size comparison task with scalar adjectives, participants included in the analysis made 7.08% errors in the whole experiment on average (min. 2%, max 14%). Data cleaning procedure resulted in exclusion of RTs of 6.59% of trials of interest (excluded incorrect responses are also counted here). The resulting mean RTs and error rates per congruity overall and in each decision polarity are given in Table 10. Mean RTs and error rates per adjective pair across decision polarities are given in Table 11.

**Table 10.** Mean RT (*SD*), error rate overall and for each decision polarity in Experiment 2b, physical size comparison with scalar adjectives.

Decision polarity	Congruent	Incongruent
Overall	761 (238) ms, 3.39%	787 (248) ms, 7.56%
‘Choose larger’	744 (232) ms, 2.78%	769 (246) ms, 7.21%
‘Choose smaller’	779 (244) ms, 4.00%	806 (250) ms, 7.92%

**Table 11.** Mean RT (*SD*), error rate per adjective pair (both decision polarities) in Experiment 2b, physical size comparison with scalar adjectives.

Adjective pair	Congruent	Incongruent
‘kort-lang’	788 (232) ms, 3.71%	789 (256) ms, 5.82%
‘laag-hoog’	730 (229) ms, 1.31%	768 (219) ms, 3.01%
‘licht-zwaar’	809 (275) ms, 7.72%	700 (189) ms, 1.91%
‘dun-dik’	714 (207) ms, 1.41%	896 (299) ms, 21.29 %
‘stil-luid’	771 (235) ms, 2.81%	812 (244) ms, 5.82%

The model with maximal random effect structure for Experiment 2b resulted in a singular fit. It was not possible to achieve a non-singular converging fit without drastically simplifying the random effect structure (which we believe would not be justified in our case since we know there must be some variability by-participant and by-item). For this reason, we examined the fit of the model with maximal random structure even though it resulted in a singular fit. None of the effects were significant (main effect of congruity - β = 30, *SE* = 50.7, *t* = 0.60, *p* = .58; main effect of decision polarity - β = 36, *SE* = 18.0, *t* = 2.01, *p* = .061; interaction of congruity and decision polarity - β = 0.8, *SE* = 10.3, *t* = 0.07, *p* = 93). The pattern that we observed here is parallel to the one observed in Experiment 1b. Whereas the mean reaction times in the congruent and congruent conditions differ in the expected direction, this difference is not consistently present for each of the adjective pairs (as can be seen in Table 11). This is reflected in a non-significant effect in the LME model.

The Bayesian LME model estimated for the main effect of congruity $\hat{\beta }$ = 18 ms, 95% CrI = [-44.63 79.09], BF_01_ = 2.8; for the main effect of decision polarity $\hat{\beta }$ = 20 ms, 95% CrI = [-14.66 54.53], BF_01_ = 2.49, for the interaction between congruity and decision polarity $\hat{\beta }$ = 1 ms, 95% CrI = [-17.83 20.39], BF_01_ = 13.34. Thus, there is no clear evidence for or against the congruity effect being zero, no clear evidence for or against the decision polarity effect being zero and strong evidence that the interaction between congruity and decision polarity is zero.

### Interim discussion

The pattern of effects that we observe in Experiments 2a and 2b is parallel to what we observed for number words (Experiment 1a and 1b). In the semantic comparison task, we observed a congruity effect. This congruity effect was present for both decision polarities (there was moderate evidence that the effect of interaction of decision polarity and congruity is zero).

Before we investigate its source in a same/different task, we need to consider the alternative explanation in terms of discriminability differences. Recall that in Experiments 2a and 2b we were able to match the stimuli in terms of variability, but not in terms of discriminability. It is possible that the size magnitude interfered with numerical magnitude processing simply because it was more salient. If this was the case, the adjective pairs with a clearer discriminability should have resulted in a larger congruity effect. However, this does not seem to be the case when inspecting the means informally: the adjective pair with the largest difference in discriminability (“stil-luid,” size comparison 110 ms faster than adjective comparison) resulted in the smallest congruity effect (the difference in the means just 4 ms). To explore this possibility formally, we re-ran the converging non-singular frequentist LME model described above additionally including the main effect of discriminability difference as well as interaction between discriminability difference and congruity as fixed factors. This model did not result in a significant main effect of discriminability or in an interaction between discriminability and congruity (though the congruity effect was also non-significant in this model so discriminability did explain some variance that was previously attributed to congruity). In addition, this model did not result in a better fit to the data than the original one, χ2(2) = 2.94, *p* = .22. We interpret these results as showing that discriminability difference does not clearly modulate the congruity effect. We, therefore, conclude that while discriminability difference remains a plausible reason behind part of the observed congruity effect, it is unlikely to completely account for it.

For the physical size comparison task with scalar adjectives we did not observe a congruity effect. In fact, the evidence is inconclusive (BF_01_ = 2.8) though the null hypothesis is supported by the data slightly more than the alternative hypothesis.

## Experiments 2c: same/different task with scalar adjectives

The same/different task with scalar adjectives was parallel to the same/different task with number words (Experiment 1c, above).

### Method

#### Participants

Because we had a restricted set of available participants meeting the criteria in the pool of registered users of Prolific.ac, participation in this task was open to those who had participated in the comparison task with number words (Experiment 1a for which the data were collected at an earlier point in time). These participants have not seen scalar adjective stimuli before and have not completed a task requiring them to pay attention to the physical size of stimuli, so we did not expect them to be in any way different from completely naive participants (as already discussed for participants of Experiment 1c above). Of the 49 participants included in the analyses of this experiment, 30 participants have previously completed Experiment 1a; the time interval between taking part in the two experiments was at least 13 days.

Fifty-five participants completed the experiment. Three participants were excluded from the analysis because they read the first instructions in less than 10 s. Two participants were excluded because they gave incorrect responses in more than 15% of trials. One further participant was excluded because they spent more than 30 min on the experiment. Due to a miscalculation at the data collection stage, we ended up with only 49 participants with valid datasets in this experiment instead of the planned 50. The mean age of the included participants was 25 years (*SD* = 4.7; 32 male and 17 female). On average, they took approximately 15:05 min to complete the experiment (*SD* = 02:57, min. 11, max. 29).

#### Stimuli

We constructed stimuli in a parallel way to how it is described for Experiment 1c, same/different task with number words, but used the adjective stimuli of Experiments 2a and 2b.

#### Procedure

The experimental procedure was identical to the one for Experiment 1c, same/different task with number words. The only difference was that the example items in the instructions and the practice items consisted of adjectives instead of number words.

### Results

Participants included in the analysis made 3.81% errors in the whole experiment on average (min. 1%, max 10%). Data cleaning procedure resulted in exclusion of RTs of 5.24% (excluded incorrect responses are also counted here) of congruent and incongruent trials according to the representational overlap account, i.e., in “different” trials. The mean reaction time in the congruent condition was 698 ms (*SD* = 163 ms, error rate: 2.8%), in the incongruent condition 710 ms (*SD* = 174 ms, error rate: 3.7%) and in the “same” trials it was 693 ms (*SD* = 158 ms, error rate: 6.1%). The “same” trials were not analysed. Mean RTs and error rates per number word pair in the congruent and incongruent “different” trials are given in Table 12.

**Table 12.** Mean RT (*SD*), error rate per scalar adjective pair in “different” trials in Experiment 2c, same/different task with scalar adjectives.

Adjective pair	Congruent	Incongruent
‘kort-lang’	691 (158) ms, 1.54%	683 (165) ms, 3.24%
‘laag-hoog’	702 (158) ms, 3.26%	718 (175) ms, 3.40%
‘licht-zwaar’	676 (174) ms, 1.54%	682 (173) ms, 0.86%
‘dun-dik’	714 (159) ms, 5.64%	726 (175) ms, 4.79%
‘stil-luid’	709 (166) ms, 2.40%	747 (174) ms, 6.51%

The frequentist LME model with maximal random effect structure included a main effect of congruity and allowed for varying intercepts per-item and per-participant as well as varying slopes for the congruity effect in each case. This model did not converge. The model excluding varying slopes for the congruity effect per participant converged. The congruity effect was not significant (β = 12, *SE* = 7.3, *t* = 1.76, *p* = .15). The Bayesian LME model estimated for the congruity effect $\hat{\beta }$ = 5 ms, 95% CrI = [-14.95 24.52], BF_01_ = 9.5; thus, there was moderate evidence for the congruity effect being zero.

In parallel to the exploratory analysis for the same/different data with number words (Experiment 1c), here we again explored whether there was learning effect over the course of the experiment by comparing mean reaction times in trials shown in the first as opposed to the second half of the experiment. Again, descriptively the RTs did get shorter over the course of the experiment, and the difference in the mean RTs between congruent and incongruent conditions was somewhat larger in the first half of the experiment (congruent: 706 ms [*SD* = 163], incongruent: 726 ms [*SD* = 182]) than in the second half of the experiment (congruent: 690 ms [*SD* = 163], incongruent: 695 ms [*SD`* = 164]). The frequentist LME models with a reasonable random effect structure did not converge, so we do not report frequentist LME results here. The Bayesian model showed moderate evidence that the interaction between congruity and experiment half was zero ( $\hat{\beta }$ = -4 ms, 95% CrI = [-29.04 20.18], BF_01_ = 9.38). Thus, even in the first half of the experiment there was no congruity effect.

### Interim discussion

Whereas we observed a congruity effect in the size congruity paradigm with scalar adjectives (Experiment 2a), the data from the present same/different task did not show a significant congruity effect. We found moderate evidence for the congruity effect being absent in the same/different task. Thus, we conclude that the congruity effect in the semantic comparison task with scalar adjectives was likely due to the conflict at the decision stage of processing. Combined with the results of the physical size comparison task with scalar adjectives (Experiment 2b), the present series of experiments does not show evidence for recruitment of GMS representations during the processing of scalar adjectives.

## General discussion

In the present project, we put forward the hypothesis that scalar adjectives such as “tall,” ‘short “long,” ‘big “loud,” etc. are symbolic references to GMS representations, and that our language comprehension system recruits GMS representations when processing these adjectives. Consistent with the observed properties of the representation format of GMS, scalar adjectives refer to only approximate values and their applicability as descriptions of magnitude depends on relative rather than absolute values. While it has been suggested in the past that processing numerals (e.g., Arabic digits or number words) recruit GMS representations, as far as we know, no research has previously looked at the potential connection of scalar adjectives and GMS representations. We compared processing of scalar adjectives to processing of number words because number words are similar to scalar adjectives in their reference to magnitude information, and the relationship of number words to GMS has previously been investigated.

In Experiments 1a and 1b, we used the size congruity paradigm with number words. The size congruity paradigm has been used in the past to look at the interaction of magnitudes evoked by number symbols and by physical size magnitude. We observed a clear congruity effect in a semantic comparison task (i.e., where numerical magnitude was the task-relevant and physical size magnitude was the task-irrelevant dimension, Experiment 1a). There was no significant congruity effect when the task-relevant and task-irrelevant dimensions were reversed (Experiment 1b). In Experiment 1c, we used the same/different paradigm with the same stimuli as in Experiments 1a and 1b. This paradigm allows to eliminate the potential conflict at the decision stage of processing as the origin of the congruity effect that we observed in the semantic comparison task. With the same/different task, we no longer observed a significant congruity effect; in fact, we had strong statistical evidence that there was no hint of a congruity effect. We thus conclude that the congruity effect that we observed with number words in the numerical comparison task was presumably primarily driven by the response conflict at the decision stage. In summary, the experiments on number words do not provide evidence that the comparison of numerical magnitudes carried by number words recruits GMS representations. The implications of this result for research on number symbol processing is discussed below.

The reasoning behind and the design of Experiments 2a-c on scalar adjectives were parallel to the Experiments 1a to 1c on the number words. Here, we again observed a congruity effect only in the semantic comparison task (participants compared the meaning of antonymous pairs of scalar adjectives and physical size magnitude was the task-irrelevant dimension), and again a congruity effect was no longer present in the same/different task with the same stimuli. Thus, as for number words, the congruity effect was primarily driven by a response conflict in the decision stage. The results of the present series of experiments thus do not provide support for the hypothesis that GMS is recruited in the processing of scalar adjective. This in turn either means that GMS is not involved in processing of scalar adjectives at all, or it implies that the size congruity paradigm is not suited to demonstrate the involvement of GMS in the processing of scalar adjectives.

### Implications of the present results for number symbol processing

As discussed in the *Introduction*, to our knowledge, the size congruity paradigm has previously been used to look at number word processing (i.e., not digits) in only three studies (Cohen Kadosh et al., 2008; Foltz et al., 1984; Ito & Hatta, 2003). A significant congruity effect in the numerical comparison task has been observed in all three studies and, in line with these studies, also in Experiment 1a of the present study. Our results thus add to this evidence in a new language—Dutch. In addition, the stimuli in our study were matched in terms of discriminability in the task-relevant and task-irrelevant dimensions. Finally, our study was the first one to administer the “choose smaller” decision polarity with number words, and we show that the congruity effect is identical for this decision polarity. Recall that the exact locus of the congruity effect in the numerical comparison task with number words has not been addressed in the past. The results of the same/different task (Experiment 2c) strongly suggest that the congruity effect is primarily driven by a response conflict in a decision stage. This implies that the decision stage could also have been the primary source for the congruity effects in these past studies as well.^6^

Two previous studies that administered a physical size comparison task with number words did not observe a significant congruity effect (in line with our Experiment 2b) while observing a congruity effect in the same task with Arabic digits or Kanji numerals (Kanji is an ideographic script; (Cohen Kadosh et al., 2008; Ito & Hatta, 2003 Experiment 1) or observed what appeared to be a qualitatively different congruity effect (Cohen Kadosh et al., 2008 Experiment 4). Ito and Hatta interpret their results as suggesting that number words do not have a strong automatic connection to the numerical magnitude representations in general or at least in case of a “less attentive processing condition” (such as when the numerical magnitude is the task-irrelevant dimension). Cohen Kadosh and colleagues go even further and argue that our cognitive system has separate comparison mechanisms for number words and Arabic numbers, and that the numerical magnitude representations of the two notations are potentially distinct, though highly interconnected. These previous studies, however, did not fully match the discriminability and variability of the stimuli (Algom et al., 1996; Pansky & Algom, 1999) along the numerical and physical size dimensions. In our physical size comparison task, the discriminability and variability *were* matched, but we still failed to observe a congruity effect. The evidence for or against the congruity effect in this task was inconclusive, but still the data was more consistent with the possibility that the congruity effect was zero (BF_01_ = 2.2). While we did not investigate Arabic digits in our own study, the results of the present experiment are consistent with the interpretations of Ito and Hatta and Cohen Kadosh and colleagues.

In summary, the present study shows that the size congruity paradigm does not provide support for the hypothesis that number words recruit GMS representations.

Given this conclusion, let us now consider how informative the results from the size congruity paradigm can be regarding recruitment of GMS representations for Arabic digits (rather than number words). First, in fact the pattern of congruity effects with Arabic digits has been observed to be different from that for number words. As discussed earlier, for Arabic digits, congruity is observed for both numerical comparison and physical size comparison tasks and is differently modulated by the numerical distance (Cohen Kadosh et al., 2008; Ito & Hatta, 2003). Furthermore, the congruity effect observed with Arabic digits has in fact been observed to be modulated by the decision polarity—the congruity effect is larger for the “choose larger” instruction than for the “choose smaller” instruction (Arend & Henik, 2015; Tzelgov et al., 1992). The presence of a modulation of the congruity effect by decision polarity is usually used as an argument against an explanation of the congruity effect originating in a conflict at the decision stage (Arend & Henik, 2015). Finally, EEG studies on the size congruity effect with Arabic digits detected ERP signatures of the congruity effect at an early point after stimulus presentation (150–250 ms after onset) which can be seen as the stage when the magnitude representations are retrieved as well as a later stage which can be seen as a decision stage conflict (though it is not possible to pinpoint an exact neural source of an ERP effect; Szucs & Soltesz, 2008). Taken together, there is thus still good evidence that *in case of Arabic digits* the size congruity effect suggests some recruitment of GMS representations, i.e., that the congruity effect results from the overlap in the magnitude representations for the numerical magnitude and size magnitude.

However, suppose that it turns out that the size congruity effect for Arabic digits is not driven by the involvement of GMS, do we then have to conclude that GMS is not involved in the processing of Arabic digits either? Evidence for interaction of number symbols and GMS representations has recently been observed in a different experimental paradigm. Lourenco and colleagues (2016) observed subliminal priming effects from Arabic digits to judgements of area. In the most interesting experiment of this study (Experiment 2), two digits were presented as primes. In the critical condition, one of the prime digits was presented in white colour and the other one in black colour. The digits were followed by the presentation of an array of white and black rectangles. Participants were asked to indicate whether the sum of the surface of, for example, the white rectangles (i.e., the cumulative white area) was the same or different as the sum of the surface of the black rectangles (i.e., the cumulative black area). In “different” trials, the colour of the numerically larger prime digit could match or mismatch the colour of the rectangles with the larger cumulative area. They observed shorter RTs when the colour of the numerically larger digit matched the colour of the rectangles with the larger cumulative area in “different” trials. Because response alternatives were not aligned with the magnitude match or mismatch between primes and cumulative areas, this design excluded the possibility that decision stage conflict is responsible for this congruity effect. This study thus provides evidence for the involvement of GMS in the processing of number symbols and GMS for which the criticism directed at the size congruity paradigm does not apply.

### Implications of the present results for scalar adjective processing

The central goal of the present project was to present and evaluate the hypothesis that the human language processing system makes use of the GMS representations during the retrieval of the meaning of scalar adjectives and the construction of a mental model of the communicated information. Like number symbols, scalar adjectives could also be symbolic references to magnitude information and share a number of features with the GMS representations. We tested this hypothesis by looking at a potential interference between retrieval of scalar adjective meaning and computation of physical size magnitude carried out simultaneously. The data collected in the present project do not support this hypothesis.

Although this series of experiments did not provide support for our hypothesis, the hypothesis and its variations remain interesting and can be investigated in a number of other ways in future research. In our experiments, we looked at whether GMS is recruited in the retrieval of the meaning of scalar adjectives presented in isolation, i.e., out of context. In follow-up studies, this specific question could be investigated using a subliminal priming paradigm that previously provided strong evidence for recruitment of GMS in the case of Arabic digits (Lourenco et al., 2016). In case of Arabic digits and area size, this paradigm provided strong evidence for interference at the level of representations.

Furthermore, scalar adjectives possibly only recruit GMS representations when they are used in a phrasal context. After all, scalar adjectives can only be interpreted in a meaningful way when they are combined with specific nouns. For example, “large mouse” and “large house” refer to different sizes, so before the size that “large” refers to can be interpreted we need to know what noun it is combined with. To test this prediction, future studies should investigate GMS recruitment in a paradigm where the meaning of the adjective has to be retrieved within a phrase or a sentence.

The hypothesis we put forward also predicts that processing scalar adjectives (but not the so-called *non-gradable adjectives* which do not refer to properties along a continuous analog scale, such as “pregnant,” ‘even “dead,” etc.) should involve neuronal populations of which we know that they are involved in the processing of magnitudes from perceptual input. Specifically, the neuronal populations in the intraparietal sulcus and surrounding areas have been consistently observed to play an important role in processing magnitudes along various perceptual dimensions (e.g., Nieder, 2016; Pinel et al., 2004; Sokolowski et al., 2017). That is where a difference between the processing of scalar versus others adjectives should be seen.

The approach suggested here can also be used to look into the processing of scalar adjectives referring to non-perceptual dimensions. While here we focused on scalar adjectives describing a perceptual dimension, there are also scalar adjectives referring to more abstract properties—e.g., “easy,” ‘difficult “kind,” ‘cruel “happy,” etc. Furthermore, our discussion has focused on scalar adjectives referring to one dimension only, but there are also scalar adjectives that can be argued to refer to magnitudes along multiple dimensions simultaneously, such as “healthy,” ‘intelligent “typical,” etc. (Sassoon, 2013). These other scalar adjectives clearly play an important role in everyday language as well, and, intuitively, the neurocognitive processes behind them should overlap with those for scalar adjectives referring to perceptual dimensions. We considered one-dimensional adjectives referring to perceptual dimensions as a more basic form, a starting point which can be used to look at the processing of these other adjectives as well.

## Conclusion

The present series of experiments investigated processing of number words and of scalar adjectives both of which can be seen as symbolic references to magnitude information. We investigated whether processing of these lexical items recruits GMS representations. The data collected for number words add to the existing literature by showing that the congruity effect with number words is most likely driven by a response conflict at the decision stage of processing. A similar conclusion was reached for the processing of scalar adjectives in the size congruity paradigm. The results of the present study thus do not support the hypothesis that the processing of scalar adjectives involves GMS representations. Furthermore, the present study reveals some serious limits of the size congruity paradigm for studying the representations involved in magnitude processing as the results strongly suggest that response conflicts in a late decision stage have a strong influence on the results. However, the hypothesis we put forward about scalar adjectives is still a promising potential line of research. We made a number of suggestions for how this hypothesis can be explored in future investigations.

## Supplemental Material

sj-docx-1-qjp-10.1177_17470218211031158 – Supplemental material for Processing symbolic magnitude information conveyed by number words and by scalar adjectives Click here for additional data file. Supplemental material, sj-docx-1-qjp-10.1177_17470218211031158 for Processing symbolic magnitude information conveyed by number words and by scalar adjectives by Arnold R Kochari and Herbert Schriefers in Quarterly Journal of Experimental Psychology

sj-docx-2-qjp-10.1177_17470218211031158 – Supplemental material for Processing symbolic magnitude information conveyed by number words and by scalar adjectives Click here for additional data file. Supplemental material, sj-docx-2-qjp-10.1177_17470218211031158 for Processing symbolic magnitude information conveyed by number words and by scalar adjectives by Arnold R Kochari and Herbert Schriefers in Quarterly Journal of Experimental Psychology

sj-docx-3-qjp-10.1177_17470218211031158 – Supplemental material for Processing symbolic magnitude information conveyed by number words and by scalar adjectives Click here for additional data file. Supplemental material, sj-docx-3-qjp-10.1177_17470218211031158 for Processing symbolic magnitude information conveyed by number words and by scalar adjectives by Arnold R Kochari and Herbert Schriefers in Quarterly Journal of Experimental Psychology

## Notes

1. Note that automaticity of access of numerical (and size) magnitude in general is a prominent line of research of its own (e.g., Dadon & Henik, 2017; Dehaene & Akhavein, 1995; Ford & Reynolds, 2016; Pansky & Algom, 2002; Wong & Szücs, 2013). Here, we are only concerned with automaticity specifically in the sense of processing of the magnitude of the irrelevant dimension in the set-up of the size congruity paradigm.

2. Furthermore, they used only two values of physical size (one large and one small), whereas 5 different number pairs were used. This means that the variability of the stimuli was larger for the numerical dimension than for the physical size dimension. However, the stark difference in the discriminability likely made the physical size considerably more salient.

3. In the norming study, we collected data for 12 different number word pairs (total number of possible number word pairs in Dutch with equal length of words within a pair) as well as 21 different font size combinations. However, we were able to match RTs of physical size comparison and number word comparison only in case of five items. That is because the general speed of comparison was substantially faster for physical size comparisons than for numerical magnitude comparisons. See the Open Science Framework website: https://osf.io/kh6eb/ for RTs and error rates for all number word pairs and font sizes that we tested. Note that in order to achieve longer RTs in the physical size comparisons we would have to make the difference in the fonts smaller than the ones we already tested, but this was not possible since smaller differences in font sizes are at some point not clearly visible anymore and result in unacceptably high error rates. We compensate for the low number of different stimulus pairs by administering a large number of trials per participant.

4. Specifically R version 3.6.3 was used along with the following packages: *brms* (version 2.12.0; Bürkner, 2017, 2018); *ggplot2* (version 3.3.0; Wickham, 2016); *Hmisc* (version 4.4-0; Harrell Jr, 2020); *knitr* (version 1.28; Xie, 2014); *lme4* (version 1.1-21; Bates et al., 2015); *lmerTest* (version 3.1-1; Kuznetsova et al., 2017); *Matrix* (version 1.2-18; Bates & Maechler, 2019); *plyr* (version 1.8.6; Wickham, 2011); *Rcpp* (version 1.0.4; Eddelbuettel & Balamuta, 2018) *readr* (version 1.3.1; Wickham et al., 2018).

5. An additional recommended criterion of convergence is effective sample sizes of at least 10% of the total number of post warm-up samples (Vasishth et al., 2018). This was not the case for one of the parameters in most of the models that we fit. Specifically, for the correlation between congruity and decision polarity slopes by participant the lowest number was 6%. However, the Rhat values were 1.01 and the effective sample size increased linearly with increasing number of iterations. Therefore, we concluded that the mixing was sufficient (Brief Guide to Stan’s Warnings, 2020).

6. Note, however, that the account of the conflict at the decision stage by Santens and colleagues (Santens & Verguts, 2011) that we have discussed in the *Introduction* cannot fully account for the results we observed in the present study either. That is because it suggests that a congruity effect should be observed regardless of which exact dimension is task-relevant and which is task-irrelevant, as long as they have two compatible response options that can compete with each other. If that were the case, we should have observed a congruity effect in the physical size comparison task as well, but we in fact did not observe a congruity effect there. Thus, additional assumptions are needed to account for the full range of the observations (we will not provide a solution here).

## References

[bibr1-17470218211031158] Algom D. Dekel A. Pansky A. (1996). The perception of number from the separability of the stimulus: The stroop effect revisited. Memory & Cognition, 24(5), 557–572. 10.3758/BF03201083 8870527

[bibr2-17470218211031158] Anobile G. Cicchini G. M. Burr D. C. (2016). Number as a primary perceptual attribute: A review. Perception, 45(1–2), 5–31. 10.1177/0301006615602599 26562858 PMC5040510

[bibr3-17470218211031158] Arend I. Henik A. (2015). Choosing the larger versus choosing the smaller: Asymmetries in the size congruity effect. Journal of Experimental Psychology: Learning, Memory, and Cognition, 41(6), 1821–1830. 10.1037/xlm0000135 26030630

[bibr4-17470218211031158] Bar H. Fischer M. H. Algom D. (2019). On the linear representation of numbers: Evidence from a new two-numbers-to-two positions task. Psychological Research, 83(1), 48–63. 10.1007/s00426-018-1063-y 30073407

[bibr5-17470218211031158] Barr D. J. Levy R. Scheepers C. Tily H. J. (2013). Random effects structure for confirmatory hypothesis testing: Keep it maximal. Journal of Memory and Language, 68(3), 255–278. 10.1016/j.jml.2012.11.001 PMC3881361 24403724

[bibr6-17470218211031158] Barth H. Kanwisher N. Spelke E. (2003). The construction of large number representations in adults. Cognition, 86(3), 201–221. 10.1016/S0010-0277(02)00178-6 12485738

[bibr7-17470218211031158] Bates D. Mächler M. Bolker B. Walker S. (2015). Fitting linear mixed-effects models using lme4. Journal of Statistical Software, 67(1). 10.18637/jss.v067.i01

[bibr8-17470218211031158] Bates D. Maechler M. (2019). Matrix: Sparse and dense matrix classes and methods. https://rdrr.io/rforge/Matrix/#:~:text=A%20rich%20hierarchy%20of%20matrix,’%20and%20’SuiteSparse’%20libraries.

[bibr9-17470218211031158] Besner D. Coltheart M. (1979). Ideographic and alphabetic processing in skilled reading of English. Neuropsychologia, 17(5), 467–472. 10.1016/0028-3932(79)90053-8 514483

[bibr10-17470218211031158] Brief Guide to Stan’s Warnings. (2020). https://mc-stan.org/misc/warnings.html#tail-ess

[bibr11-17470218211031158] Buckley P. B. Gillman C. B. (1974). Comparisons of digits and dot patterns. Journal of Experimental Psychology, 103(6), 1131–1136. 10.1037/h0037361 4457588

[bibr12-17470218211031158] Bulthé J. De Smedt B. Op de Beeck H. (2014). Format-dependent representations of symbolic and non-symbolic numbers in the human cortex as revealed by multi-voxel pattern analyses. NeuroImage, 87, 311–322. 10.1016/J.NEUROIMAGE.2013.10.049 24201011

[bibr13-17470218211031158] Bulthé J. Smedt B. D. de Beeck H. P. O. (2015). Visual number beats abstract numerical magnitude: Format-dependent representation of Arabic digits and dot patterns in human parietal cortex. Journal of Cognitive Neuroscience, 27(7), 1376–1387. 10.1162/jocn_a_00787 25633646

[bibr14-17470218211031158] Bürkner P.-C. (2017). brms: An R package for Bayesian multilevel models using Stan. Journal of Statistical Software, 80(1), 1–28. 10.18637/jss.v080.i01

[bibr15-17470218211031158] Bürkner P.-C. (2018). Advanced Bayesian multilevel modeling with the R Package brms. The R Journal, 10(1), 395–411.

[bibr16-17470218211031158] Campbell J. I. D. Epp L. J. (2004). An encoding-complex approach to numerical cognition in Chinese-English Bilinguals. Canadian Journal of Experimental Psychology/Revue canadienne de psychologie expérimentale, 58(4), 229–244. 10.1037/h0087447 15648727

[bibr17-17470218211031158] Cantlon J. F. Brannon E. M. Carter E. J. Pelphrey K. A. (2006). Functional imaging of numerical processing in adults and 4-y-old children. PLOS Biology, 4(5), Article e125. 10.1371/journal.pbio.0040125 PMC1431577 16594732

[bibr18-17470218211031158] Cantlon J. F. Platt M. L. Brannon E. M. (2009). Beyond the number domain. Trends in Cognitive Sciences, 13(2), 83–91. 10.1016/j.tics.2008.11.007 19131268 PMC2709421

[bibr19-17470218211031158] Cipolotti L. Butterworth B. (1995). Toward a multiroute model of number processing: Impaired number transcoding with preserved calculation skills. Journal of Experimental Psychology: General, 124(4), 375–390.

[bibr20-17470218211031158] Clementz B. A. Barber S. K. Dzau J. R. (2002). Knowledge of stimulus repetition affects the magnitude and spatial distribution of low-frequency event-related brain potentials. Audiology and Neurotology, 7(5), 303–314. 10.1159/000064444 12232500

[bibr21-17470218211031158] Cohen Kadosh R. Cohen Kadosh K. Linden D. E. J. Gevers W. Berger A. Henik A . (2007). The brain locus of interaction between number and size: A combined functional magnetic resonance imaging and event-related potential study. Journal of Cognitive Neuroscience, 19(6), 957–970. 10.1162/jocn.2007.19.6.957 17536966

[bibr22-17470218211031158] Cohen Kadosh R. Henik A. Rubinsten O . (2008). Are Arabic and verbal numbers processed in different ways? Journal of Experimental Psychology: Learning, Memory, and Cognition, 34(6), 1377–1391. 10.1037/a0013413 18980402

[bibr23-17470218211031158] Cohen Kadosh R. Lammertyn J. Izard V . (2008). Are numbers special? An overview of chronometric, neuroimaging, developmental and comparative studies of magnitude representation. Progress in Neurobiology, 84(2), 132–147. 10.1016/j.pneurobio.2007.11.001 18155348

[bibr24-17470218211031158] Crump M. J. C. McDonnell J. V. Gureckis T. M. (2013). Evaluating Amazon’s mechanical turk as a tool for experimental behavioral research. PLOS ONE, 8(3), Article e57410. 10.1371/journal.pone.0057410 PMC3596391 23516406

[bibr25-17470218211031158] Dadon G. Henik A. (2017). Adjustment of control in the numerical Stroop task. Memory & Cognition, 45(6), 891–902. 10.3758/s13421-017-0703-6 28337604 PMC5529565

[bibr26-17470218211031158] Defever E. Sasanguie D. Gebuis T. Reynvoet B. (2011). Children’s representation of symbolic and nonsymbolic magnitude examined with the priming paradigm. Journal of Experimental Child Psychology, 109(2), 174–186. 10.1016/j.jecp.2011.01.002 21324472

[bibr27-17470218211031158] Dehaene S. (1992). Varieties of numerical abilities. Cognition, 44(1), 1–42. 10.1016/0010-0277(92)90049-N 1511583

[bibr28-17470218211031158] Dehaene S. (1997). The number sense. How the mind creates mathematics. Oxford University Press. 10.2307/2589308

[bibr29-17470218211031158] Dehaene S. (2007). Symbols and quantities in parietal cortex: Elements of a mathematical theory of number representation and manipulation. In Haggard P. Rosetti Y. Kawato M. (Eds.), Sensorimotor foundations of higher cognition (pp. 527–574). Oxford University Press.

[bibr30-17470218211031158] Dehaene S. Akhavein R. (1995). Attention, automaticity, and levels of representation in number processing. Journal of Experimental Psychology: Learning, Memory, and Cognition, 21(2), 314–326. 10.1037/0278-7393.21.2.314 7738503

[bibr31-17470218211031158] Dehaene S. Cohen L. (2007). Cultural recycling of cortical maps. Neuron, 56(2), 384–398. 10.1016/j.neuron.2007.10.004 17964253

[bibr32-17470218211031158] Dehaene S. Izard V. Spelke E. Pica P. (2008). Log or linear? Distinct intuitions of the number scale in Western and Amazonian indigene cultures. Science, 320(5880), 1217–1220. 10.1126/science.1156540 18511690 PMC2610411

[bibr33-17470218211031158] de Leeuw J. R . (2015). jsPsych: A JavaScript library for creating behavioral experiments in a Web browser. Behavior Research Methods, 47(1), 1–12. 10.3758/s13428-014-0458-y 24683129

[bibr34-17470218211031158] de Leeuw J. R. Motz B. A . (2016). Psychophysics in a Web browser? Comparing response times collected with JavaScript and Psychophysics Toolbox in a visual search task. Behavior Research Methods, 48, 1–12. 10.3758/s13428-015-0567-2 25761390

[bibr35-17470218211031158] Dickey J. M. Lientz B. P. (1970). The weighted likelihood ratio, sharp hypotheses about chances, the order of a Markov chain. The Annals of Mathematical Statistics, 41(1), 214–226. 10.1214/aoms/1177697203

[bibr36-17470218211031158] Eddelbuettel D. Balamuta J. J. (2018). Extending R with C++: A brief introduction to Rcpp. The American Statistician, 72(1), 28–36. 10.1080/00031305.2017.1375990

[bibr37-17470218211031158] Eger E . (2016). Neuronal foundations of human numerical representations. In Cappelletti M. Fias W. (Eds.), Progress in brain research (Vol. 227, pp. 1–27). Elsevier. 10.1016/bs.pbr.2016.04.015 27339006

[bibr38-17470218211031158] Faulkenberry T. J. Cruise A. Lavro D. Shaki S. (2016). Response trajectories capture the continuous dynamics of the size congruity effect. Acta Psychologica, 163, 114–123. 10.1016/j.actpsy.2015.11.010 26647112

[bibr39-17470218211031158] Feigenson L. Dehaene S. Spelke E. (2004). Core systems of number. Trends in Cognitive Sciences, 8(7), 307–314. 10.1016/j.tics.2004.05.002 15242690

[bibr40-17470218211031158] Foltz G. S. Poltrock S. E. Potts G. R. (1984). Mental comparison of size and magnitude: Size congruity effects. Journal of Experimental Psychology: Learning, Memory, and Cognition, 10(3), 442–453. 10.1037/0278-7393.10.3.442 6235311

[bibr41-17470218211031158] Ford N. Reynolds M. G. (2016). Do Arabic numerals activate magnitude automatically? Evidence from the psychological refractory period paradigm. Psychonomic Bulletin & Review, 23(5), 1528–1533. 10.3758/s13423-016-1020-y 27025502

[bibr42-17470218211031158] Frazier L. Clifton C. Stolterfoht B. (2008). Scale structure: Processing minimum standard and maximum standard scalar adjectives. Cognition, 106(1), 299–324. 10.1016/J.COGNITION.2007.02.004 17376422 PMC2259288

[bibr43-17470218211031158] Fults S. (2011). Vagueness and scales. In Égré P. Klinedinst N. (Eds.), Vagueness and language use (pp. 25–50). Palgrave Macmillan UK.

[bibr44-17470218211031158] Gabay S. Leibovich T. Henik A. Gronau N. (2013). Size before numbers: Conceptual size primes numerical value. Cognition, 129(1), 18–23. 10.1016/j.cognition.2013.06.001 23811178

[bibr45-17470218211031158] Gallistel C. R. Gelman R. (1992). Preverbal and verbal counting and computation. Cognition, 44(1), 43–74. 10.1016/0010-0277(92)90050-R 1511586

[bibr46-17470218211031158] Gallistel C. R. Gelman R. (2000). Non-verbal numerical cognition: From reals to integers. Trends in Cognitive Sciences, 4(2), 59–65. 10.1016/S1364-6613(99)01424-2 10652523

[bibr47-17470218211031158] Gebuis T. Cohen Kadosh R. Gevers W. (2016). Sensory-integration system rather than approximate number system underlies numerosity processing: A critical review. Acta Psychologica, 171, 17–35. 10.1016/j.actpsy.2016.09.003 27640140

[bibr48-17470218211031158] Goffin C. Sokolowski H. M. Slipenkyj M. Ansari D. (2019). Does writing handedness affect neural representation of symbolic number? An fMRI adaptation study. Cortex, 121, 27–43. 10.1016/j.cortex.2019.07.017 31541860

[bibr49-17470218211031158] Halberda J. Feigenson L. (2008). Developmental change in the acuity of the “Number Sense”: The approximate number system in 3-, 4-, 5-, and 6-year-olds and adults. Developmental Psychology, 44(5), 1457–1465. 10.1037/a0012682 18793076

[bibr50-17470218211031158] Harrell F. E. Jr. (2020). Hmisc: Harrell miscellaneous.

[bibr51-17470218211031158] He L. Zhou K. Zhou T. He S. Chen L. (2015). Topology-defined units in numerosity perception. Proceedings of the National Academy of Sciences, 112(41), E5647–E5655. 10.1073/pnas.1512408112 PMC4611617 26417075

[bibr52-17470218211031158] Henik A. Tzelgov J. (1982). Is three greater than five: The relation between physical and semantic size in comparison tasks. Memory & Cognition, 10(4), 389–395. 10.3758/BF03202431 7132716

[bibr53-17470218211031158] Holloway I. D. Battista C. Vogel S. E. Ansari D. (2012). Semantic and perceptual processing of number symbols: Evidence from a cross-linguistic fMRI adaptation study. Journal of Cognitive Neuroscience, 25(3), 388–400. 10.1162/jocn_a_00323 23163414

[bibr54-17470218211031158] Ito Y. Hatta T. (2003). Semantic processing of Arabic, Kanji, and Kana numbers: Evidence from interference in physical and numerical size judgments. Memory & Cognition, 31(3), 360–368. 10.3758/BF03194394 12795478

[bibr55-17470218211031158] Jeffreys S. H. (1998). The Theory of Probability (3rd ed.). Oxford University Press.

[bibr56-17470218211031158] Kaufmann L. Koppelstaetter F. Delazer M. Siedentopf C. Rhomberg P. Golaszewski S. Felber S. Ischebeck A. (2005). Neural correlates of distance and congruity effects in a numerical Stroop task: An event-related fMRI study. NeuroImage, 25(3), 888–898. 10.1016/j.neuroimage.2004.12.041 15808989

[bibr57-17470218211031158] Kochari A. (2019). Conducting web-based experiments for numerical cognition research. Journal of Cognition, 2(1), 39. 10.5334/joc.85 31576378 PMC6753310

[bibr58-17470218211031158] Kochari A. Lewis A. Schoffelen J.-M. Schriefers H. (2021). Semantic and syntactic composition of minimal adjective-noun phrases in Dutch: An MEG study. Neuropsychologia, 155, 107754. 10.1016/j.neuropsychologia.2021.107754 33476626

[bibr59-17470218211031158] Koechlin E. Naccache L. Block E. Dehaene S. (1999). Primed numbers: Exploring the modularity of numerical representations with masked and unmasked semantic priming. Journal of Experimental Psychology: Human Perception and Performance, 25(6), 1882–1905. 10.1037/0096-1523.25.6.1882

[bibr60-17470218211031158] Kojouharova P. Krajcsi A. (2020). The Indo-Arabic distance effect originates in the response statistics of the task. Psychological Research, 84, 468–480. 10.1007/s00426-018-1052-1 30030613

[bibr61-17470218211031158] Krajcsi A. Lengyel G. Kojouharova P. (2018). Symbolic number comparison is not processed by the analog number system: Different symbolic and non-symbolic numerical distance and size effects. Frontiers in Psychology, 9, Article 124. 10.3389/fpsyg.2018.00124 PMC5817629 29491845

[bibr62-17470218211031158] Kuznetsova A. Brockhoff P. B. Christensen R. H. B. (2017). lmerTest package: Tests in linear mixed effects models. Journal of Statistical Software, 82(13), 1–26. 10.18637/jss.v082.i13

[bibr63-17470218211031158] Leibovich T. Diesendruck L. Rubinsten O. Henik A. (2013). The importance of being relevant: Modulation of magnitude representations. Frontiers in Psychology, 4, 369. 10.3389/fpsyg.2013.00369 23805117 PMC3693217

[bibr64-17470218211031158] Leibovich T. Katzin N. Harel M. Henik A. (2017). From “sense of number” to ‘sense of magnitude’: The role of continuous magnitudes in numerical cognition. Behavioral and Brain Sciences, 40, e164. 10.1017/S0140525X16000960 27530053

[bibr65-17470218211031158] Lindeløv J. K. (2020). Reaction time distributions: An interactive overview. https://lindeloev.shinyapps.io/shiny-rt/

[bibr66-17470218211031158] Lourenco S. F. (2015). On the relation between numerical and non-numerical magnitudes: Evidence for a general magnitude system. In Geary D. C. Berch D. B. Koepke K. M. (Eds.), Evolutionary origins and early development of number processing (Vol. 1, pp. 145–174). Elsevier. 10.1016/B978-0-12-420133-0.00006-5

[bibr67-17470218211031158] Lourenco S. F. Ayzenberg V. Lyu J. (2016). A general magnitude system in human adults: Evidence from a subliminal priming paradigm. Cortex, 81, 93–103. 10.1016/j.cortex.2016.04.013 27179917

[bibr68-17470218211031158] Lyons I. M. Ansari D. Beilock S. L. (2015). Qualitatively different coding of symbolic and nonsymbolic numbers in the human brain. Human Brain Mapping, 36(2), 475–488. 10.1002/hbm.22641 25238646 PMC6869776

[bibr69-17470218211031158] Lyons I. M. Beilock S. L. (2018). Characterizing the neural coding of symbolic quantities. NeuroImage, 178, 503–518. 10.1016/j.neuroimage.2018.05.062 29857048

[bibr70-17470218211031158] McCloskey M. (1992). Cognitive mechanisms in numerical processing: Evidence from acquired dyscalculia. Cognition, 44(1), 107–157. 10.1016/0010-0277(92)90052-J 1511584

[bibr71-17470218211031158] Merten K. Nieder A. (2008). Compressed scaling of abstract numerosity representations in adult humans and monkeys. Journal of Cognitive Neuroscience, 21(2), 333–346. 10.1162/jocn.2008.21032 18510443

[bibr72-17470218211031158] Moyer R. S. Landauer T. K. (1967). Time required for judgements of numerical inequality. Nature, 215(5109), 1519–1520. 10.1038/2151519a0 6052760

[bibr73-17470218211031158] Nalborczyk L. Batailler C. Lœ venbruck H. Vilain A. Bürkner P.-C. (2019). An introduction to Bayesian multilevel models using brms: A case study of gender effects on vowel variability in standard Indonesian. Journal of Speech, Language, and Hearing Research, 62(5), 1225–1242. 10.1044/2018_JSLHR-S-18-0006 31082309

[bibr74-17470218211031158] Nicenboim B. Vasishth S. (2016). Statistical methods for linguistic research: Foundational Ideas—Part II. Language and Linguistics Compass, 10(11), 591–613. 10.1111/lnc3.12207

[bibr75-17470218211031158] Nieder A. (2016). The neuronal code for number. Nature Reviews Neuroscience, 17(6), 366–382. 10.1038/nrn.2016.40 27150407

[bibr76-17470218211031158] Palan S. Schitter C. (2018). Prolific.ac—A subject pool for online experiments. Journal of Behavioral and Experimental Finance, 17, 22–27. 10.1016/j.jbef.2017.12.004

[bibr77-17470218211031158] Pansky A. Algom D. (1999). Stroop and Garner effects in comparative judgment of numerals: The role of attention. Journal of Experimental Psychology: Human Perception and Performance, 25(1), 39–58.

[bibr78-17470218211031158] Pansky A. Algom D. (2002). Comparative judgment of numerosity and numerical magnitude: Attention preempts automaticity. Journal of Experimental Psychology: Learning, Memory, and Cognition, 28(2), 259–274. 10.1037/0278-7393.28.2.259 11911383

[bibr79-17470218211031158] Piazza M. Eger E. (2016). Neural foundations and functional specificity of number representations. Neuropsychologia, 83, 257–273. 10.1016/j.neuropsychologia.2015.09.025 26403660

[bibr80-17470218211031158] Piazza M. Izard V. Pinel P. Le Bihan D. Dehaene S. (2004). Tuning curves for approximate numerosity in the human intraparietal sulcus. Neuron, 44(3), 547–555. 10.1016/j.neuron.2004.10.014 15504333

[bibr81-17470218211031158] Pica P. Lemer C. Izard V. Dehaene S. (2004). Exact and approximate arithmetic in an Amazonian indigene group. Science, 306(5695), 499–503. 10.1126/science.1102085 15486303

[bibr82-17470218211031158] Pinel P. Piazza M. Le Bihan D. Dehaene S. (2004). Distributed and overlapping cerebral representations of number, size, and luminance during comparative judgments. Neuron, 41(6), 983–993. 10.1016/S0896-6273(04)00107-2 15046729

[bibr83-17470218211031158] Proctor R. W. Cho Y. S. (2006). Polarity correspondence: A general principle for performance of speeded binary classification tasks. Psychological Bulletin, 132(3), 416–442. 10.1037/0033-2909.132.3.416 16719568

[bibr84-17470218211031158] R Core Team. (2020). R: A language and environment for statistical computing. R Foundation for Statistical Computing.

[bibr85-17470218211031158] Reike D. Schwarz W. (2017). Exploring the origin of the number-size congruency effect: Sensitivity or response bias? Attention, Perception, & Psychophysics, 79(2), 383–388. 10.3758/s13414-016-1267-4 28130715

[bibr86-17470218211031158] Reimers S. Stewart N. (2015). Presentation and response timing accuracy in Adobe Flash and HTML5/JavaScript Web experiments. Behavior Research Methods, 47(2), 309–327. 10.3758/s13428-014-0471-1 24903687 PMC4427652

[bibr87-17470218211031158] Reynvoet B. De Smedt B. Van den Bussche E. (2009). Children’s representation of symbolic magnitude: The development of the priming distance effect. Journal of Experimental Child Psychology, 103(4), 480–489. 10.1016/j.jecp.2009.01.007 19285684

[bibr88-17470218211031158] Risko E. F. Maloney E. A. Fugelsang J. A. (2013). Paying attention to attention: Evidence for an attentional contribution to the size congruity effect. Attention, Perception, & Psychophysics, 75(6), 1137–1147. 10.3758/s13414-013-0477-2 23709065

[bibr89-17470218211031158] Roggeman C. Verguts T. Fias W. (2007). Priming reveals differential coding of symbolic and non-symbolic quantities. Cognition, 105(2), 380–394. 10.1016/j.cognition.2006.10.004 17125760

[bibr90-17470218211031158] Rousselet G. A. Wilcox R. R. (2019). Reaction times and other skewed distributions: Problems with the mean and the median. bioRxiv, 383935. 10.1101/383935

[bibr91-17470218211031158] Santens S. Verguts T. (2011). The size congruity effect: Is bigger always more? Cognition, 118(1), 94–110. 10.1016/j.cognition.2010.10.014 21074146

[bibr92-17470218211031158] Sasanguie D. Defever E. Van den Bussche E. Reynvoet B. (2011). The reliability of and the relation between non-symbolic numerical distance effects in comparison, same-different judgments and priming. Acta Psychologica, 136(1), 73–80. 10.1016/j.actpsy.2010.10.004 21075357

[bibr93-17470218211031158] Sasanguie D. De Smedt B. Reynvoet B. (2017). Evidence for distinct magnitude systems for symbolic and non-symbolic number. Psychological Research, 81(1), 231–242. 10.1007/s00426-015-0734-1 26708496

[bibr94-17470218211031158] Sassoon G. W. (2013). A typology of multidimensional adjectives. Journal of Semantics, 30(3), 335–380. 10.1093/jos/ffs012

[bibr95-17470218211031158] Schwarz W. Heinze H.-J. (1998). On the interaction of numerical and size information in digit comparison: A behavioral and event-related potential study. Neuropsychologia, 36(11), 1167–1179. 10.1016/S0028-3932(98)00001-3 9842762

[bibr96-17470218211031158] Sedivy J. C. Tanenhaus M. K. Chambers C. G. Carlson G. N. (1999). Achieving incremental semantic interpretation through contextual representation. Cognition, 71(2), 109–147. 10.1016/S0010-0277(99)00025-6 10444906

[bibr97-17470218211031158] Semmelmann K. Weigelt S. (2017). Online psychophysics: Reaction time effects in cognitive experiments. Behavior Research Methods, 49(4), 1241–1260. 10.3758/s13428-016-0783-4 27496171

[bibr98-17470218211031158] Sokolowski H. M. Ansari D. (2016). Symbolic and nonsymbolic representation of number in the human parietal cortex: A review of the state-of-the-art, outstanding questions and future directions. In Henik A. (Ed.), Continuous issues in numerical cognition (pp. 326–353). Academic Press. 10.1016/B978-0-12-801637-4.00015-9

[bibr99-17470218211031158] Sokolowski H. M. Fias W. Bosah Ononye C. Ansari D. (2017). Are numbers grounded in a general magnitude processing system? A functional neuroimaging meta-analysis. Neuropsychologia, 105, 50–69. 10.1016/j.neuropsychologia.2017.01.019 28119003

[bibr100-17470218211031158] Solt S. (2015). Vagueness and imprecision: Empirical foundations. Annual Review of Linguistics, 1(1), 107–127. 10.1146/annurev-linguist-030514-125150

[bibr101-17470218211031158] Szucs D. Soltesz F. (2008). The interaction of task-relevant and task-irrelevant stimulus features in the number/size congruency paradigm: An ERP study. Brain Research, 1190(1), 143–158. 10.1016/j.brainres.2007.11.010 18076868

[bibr102-17470218211031158] Tzelgov J. Meyer J. Henik A. (1992). Automatic and intentional processing of numerical information. Journal of Experimental Psychology: Learning, Memory, and Cognition, 18(1), 166–179. 10.1037/0278-7393.18.1.166

[bibr103-17470218211031158] Van Opstal F. Gevers W. De Moor W. Verguts T . (2008). Dissecting the symbolic distance effect: Comparison and priming effects in numerical and nonnumerical orders. Psychonomic Bulletin & Review, 15(2), 419–425. 10.3758/PBR.15.2.419 18488662

[bibr104-17470218211031158] Van Opstal F. Verguts T . (2011). The origins of the numerical distance effect: The same–different task. Journal of Cognitive Psychology, 23(1), 112–120. 10.1080/20445911.2011.466796

[bibr105-17470218211031158] van Rooij R . (2011). Vagueness and linguistics. In Vagueness: A guide (pp. 123–170). Springer Netherlands. 10.1007/978-94-007-0375-9_6

[bibr106-17470218211031158] Vasishth S. Nicenboim B. Beckman M. E. Li F. Kong E. J. (2018). Bayesian data analysis in the phonetic sciences: A tutorial introduction. Journal of Phonetics, 71, 147–161. 10.1016/j.wocn.2018.07.008 30197458 PMC6124675

[bibr107-17470218211031158] Verguts T. Fias W. Stevens M. (2005). A model of exact small-number representation. Psychonomic Bulletin & Review, 12(1), 66–80. 10.3758/BF03196349 15945201

[bibr108-17470218211031158] Vogel S. E. Goffin C. Bohnenberger J. Koschutnig K. Reishofer G. Grabner R. H. Ansari D. (2017). The left intraparietal sulcus adapts to symbolic number in both the visual and auditory modalities: Evidence from fMRI. NeuroImage, 153, 16–27. 10.1016/j.neuroimage.2017.03.048 28341165

[bibr109-17470218211031158] Wagenmakers E.-J. Lodewyckx T. Kuriyal H. Grasman R. (2010). Bayesian hypothesis testing for psychologists: A tutorial on the Savage–Dickey method. Cognitive Psychology, 60(3), 158–189. 10.1016/j.cogpsych.2009.12.001 20064637

[bibr110-17470218211031158] Walsh V. (2003). A theory of magnitude: Common cortical metrics of time, space and quantity. Trends in Cognitive Sciences, 7(11), 483–488. 10.1016/j.tics.2003.09.002 14585444

[bibr111-17470218211031158] Walsh V. (2015). A theory of magnitude: The parts that sum to number. In Cohen Kadosh R. Dowker A. (Eds.), The Oxford handbook of numerical cognition (pp. 552–565). Oxford University Press.

[bibr112-17470218211031158] Wickham H. (2011). The split-apply-combine strategy for data analysis. Journal of Statistical Software, 40(1), 1–29.

[bibr113-17470218211031158] Wickham H. (2016). Ggplot2: Elegant graphics for data analysis. Springer-Verlag.

[bibr114-17470218211031158] Wickham H. Hester J. Francois R. (2018). Readr: Read rectangular text data. https://readr.tidyverse.org/reference/readr-package.html

[bibr115-17470218211031158] Wilkey E. D. Ansari D. (2019). Challenging the neurobiological link between number sense and symbolic numerical abilities. Annals of the New York Academy of Sciences, 1464, 76–98.. 10.1111/nyas.14225 31549430

[bibr116-17470218211031158] Wong B. Szücs D. (2013). Single-digit Arabic numbers do not automatically activate magnitude representations in adults or in children: Evidence from the symbolic same–different task. Acta Psychologica, 144(3), 488–498. 10.1016/j.actpsy.2013.08.006 24076332 PMC3842502

[bibr117-17470218211031158] Xie Y. (2014). Knitr: A comprehensive tool for reproducible research in R. In Stodden V. Leisch F. Peng R. D. (Eds.), Implementing reproducible research (pp. 3–31). Chapman & Hall/CRC. 10.1201/9781315373461-1

[bibr118-17470218211031158] Ziegler J. Pylkkänen L. (2016). Scalar adjectives and the temporal unfolding of semantic composition: An MEG investigation. Neuropsychologia, 89, 161–171. 10.1016/j.neuropsychologia.2016.06.010 27297726

[bibr119-17470218211031158] Zwaan R. A. Pecher D. Paolacci G. Bouwmeester S. Verkoeijen P. Dijkstra K. Zeelenberg R. (2018). Participant Nonnaiveté and the reproducibility of cognitive psychology. Psychonomic Bulletin & Review, 25(5), 1968–1972. 10.3758/s13423-017-1348-y 28744765

