# Supplementary material for: Processing symbolic magnitude information conveyed by number words and by scalar adjectives
Source: Q J Exp Psychol (Hove). 2021 Jul 16;75(3):422–49. doi: 10.1177/17470218211031158 (PMC8793294; doi:10.1177/17470218211031158)
Supplement: sj-docx-1-qjp-10.1177_17470218211031158 – Supplemental material for Processing symbolic magnitude information conveyed by number words and by scalar adjectives [file sj-docx-1-qjp-10.1177_17470218211031158.docx]

**Online Supplementary Material A:
Brief overview of the discussion about existence of the generalized magnitude system**

There is a number of reasons to postulate the existence of GMS in addition to the parallel ratio-based performance across different dimensions. Specifically, a considerable amount of evidence shows transfer or interference effects between magnitude information in different dimensions (e.g., size and quantity, duration and length) when magnitudes in two dimensions have to be judged consecutively or when they are presented simultaneously and one dimension is task-irrelevant (e.g., Bonn & Cantlon, 2017; Casasanto & Boroditsky, 2008; Dormal, Seron, & Pesenti, 2006; Droit-Volet, 2010; Krause, Bekkering, Pratt, & Lindemann, 2017; Lourenco, Ayzenberg, & Lyu, 2016; Möhring, Ramsook, Hirsh-Pasek, Golinkoff, & Newcombe, 2016; Oliveri et al., 2008; Stevens, Mack, & Stevens, 1960; Xuan et al., 2007). For example, in a recent study Bonn and Cantlon (2017) observed spontaneous extraction and transfer of ratio information between e.g., size and duration or size and loudness. Specifically, when asked to judge the similarity of sequences of stimuli in two different dimensions, participants rated stimulus sequences which preserved the ratio information across dimensions as more similar than sequences which preserved only rank information across dimensions. It has also been claimed that there is a similar developmental pattern in the precision with which children are able to discriminate magnitudes in different dimensions (Feigenson, 2007). Finally, there is neuropsychological and neuroimaging evidence suggesting that potentially overlapping neural populations in the intraparietal cortex are involved in the processing magnitudes in different dimensions (e.g., Chassy & Grodd, 2012; Fias, Lammertyn, Reynvoet, Dupont, & a Orban, 2003; Pinel, Piazza, Le Bihan, & Dehaene, 2004; Sokolowski, Fias, Bosah Ononye, & Ansari, 2017; Zorzi, Priftis, & Umiltà, 2002; see also Nieder, 2016 for references to studies which observe individual neurons responsive to magnitudes in different dimensions).

It should be noted, however, that there is also an active debate about the existence of the GMS and about what exactly is shared between dimensions (for reviews and discussion, see Bonn & Cantlon, 2012; Cantlon et al., 2009; Cohen Kadosh, Lammertyn, & Izard, 2008; Leibovich et al., 2017; Lourenco, 2015; Sokolowski, Fias, Bosah Ononye, & Ansari, 2017; Van Opstal & Verguts, 2013; Yates et al., 2012). Evidence against a shared GMS comes, for example, from the observation that transfer effects between magnitudes are not always bidirectional (e.g., Bonn, 2015; Merritt, Casasanto, & Brannon, 2010; Roitman, Brannon, Andrews, & Platt, 2007). Also, the results of interference studies and neuroimaging studies could have an alternative interpretation in terms of learned associations due to co-occurrence in natural environments (see Bonn, 2015; de Hevia & Spelke, 2009; van Galen & Reitsma, 2008).

**References**

Bonn, C. D., & Cantlon, J. F. (2012). The origins and structure of quantitative concepts. *Cognitive neuropsychology*, *29*(1-2), 149–73. doi: 10.1080/02643294.2012.707122

Bonn, C. D. (2015). *On Theories of Abstract, Quantitative Representation* (PhD Thesis). University of Rochester, New York.

Bonn, C. D., & Cantlon, J. F. (2017). Spontaneous, modality-general abstraction of a ratio scale. *Cognition*, *169*, 36–45. doi: 10.1016/J.COGNITION.2017.07.012 *Brief Guide to Stan’s Warnings.* (2020). https://mc-stan.org/misc/warnings.html#tail-ess.

Casasanto, D., & Boroditsky, L. (2008). Time in the mind: Using space to think about time. *Cognition*, *106*(2), 579–593. doi: 10.1016/j.cognition.2007.03.004

Cantlon, J. F., Platt, M. L., & Brannon, E. M. (2009). Beyond the number domain. *Trends in Cognitive Sciences*, *13*(2), 83–91. doi: 10.1016/j.tics.2008.11.007

Chassy, P., & Grodd, W. (2012). Comparison of Quantities: Core and Format-Dependent Regions as Revealed by fMRI. *Cerebral Cortex*, *22*(6), 1420–1430. doi: 10.1093/cercor/bhr219

Cohen Kadosh, R., Lammertyn, J., & Izard, V. (2008). Are numbers special? An overview of chronometric, neuroimaging, developmental and comparative studies of magnitude representation. *Progress in Neurobiology*, *84*(2), 132–147. doi: 10.1016/j.pneurobio.2007.11.001

Dormal, V., Seron, X., & Pesenti, M. (2006). Numerosity-duration interference: A Stroop experiment. *Acta Psychologica*, *121*(2), 109–124. doi: 10.1016/j.actpsy.2005.06.003

Droit-Volet, S. (2010). Speeding up a master clock common to time, number and length? *Behavioural Processes*, *85*(2), 126–134. doi: 10.1016/j.beproc.2010.06.017

de Hevia, M.-D., & Spelke, E. S. (2009). Spontaneous mapping of number and space in adults and young children. *Cognition*, *110*(2), 198–207. doi: 10.1016/j.cognition.2008.11.003

Feigenson, L. (2007). The equality of quantity. *Trends in Cognitive Sciences*, *11*(5), 185–187. doi: 10.1016/j.tics.2007.01.006

Fias, W., Lammertyn, J., Reynvoet, B., Dupont, P., & a Orban, G. (2003). Parietal representation of symbolic and nonsymbolic magnitude. *Journal of cognitive neuroscience*, *15*(1), 47–56. doi: 10.1162/089892903321107819

Krause, F., Bekkering, H., Pratt, J., & Lindemann, O. (2017). Interaction between numbers and size during visual search. *Psychological Research*, *81*(3), 664–677. doi: 10.1007/s00426-016-0771-4

Leibovich, T., Katzin, N., Harel, M., & Henik, A. (2017). From ‘sense of number’ to ‘sense of magnitude’: The role of continuous magnitudes in numerical cognition. *Behavioral and Brain Sciences*, *40*, E164. doi: 10.1017/S0140525X16000960

Lourenco, S. F. (2015). On the relation between numerical and non-numerical magnitudes: Evidence for a general magnitude system. In D. C. Geary, D. B. Berch, & K. M. Koepke (Eds.), *Evolutionary origins and early development of number processing* (Vol. 1, pp. 145–174). Elsevier. doi: 10.1016/B978-0-12-420133-0.00006-5

Lourenco, S. F., Ayzenberg, V., & Lyu, J. (2016). A general magnitude system in human adults: Evidence from a subliminal priming paradigm. *Cortex*, *81*, 93–103. doi: 10.1016/j.cortex.2016.04.013

Merritt, D. J., Casasanto, D., & Brannon, E. M. (2010). Do monkeys think in metaphors? Representations of space and time in monkeys and humans. *Cognition*, *117*(2), 191–202. doi: 10.1016/j.cognition.2010.08.011

Möhring, W., Ramsook, K. A., Hirsh-Pasek, K., Golinkoff, R. M., & Newcombe, N. S. (2016). *Where music meets space: Children’s sensitivity to pitch intervals is related to their mental spatial transformation skills* (Vol. 151; Tech. Rep.). doi: 10.1016/j.cognition.2016.02.016

Nieder, A. (2016). The neuronal code for number. *Nature Reviews Neuroscience*, *17*(6), 366–382. doi: 10.1038/nrn.2016.40

Oliveri, M., Vicario, C. M., Salerno, S., Koch, G., Turriziani, P., Mangano, R., . . . Caltagirone, C. (2008). Perceiving numbers alters time perception. *Neuroscience Letters*, *438*(3), 308–311. doi: 10.1016/j.neulet.2008.04.051

Pinel, P., Piazza, M., Le Bihan, D., & Dehaene, S. (2004). Distributed and overlapping cerebral representations of number, size, and luminance during comparative judgments. *Neuron*, *41*(6), 983–993. doi: 10.1016/S0896-6273(04)00107-2

Roitman, J. D., Brannon, E. M., Andrews, J. R., & Platt, M. L. (2007). Nonverbal representation of time and number in adults. *Acta Psychologica*, *124*(3), 296–318. doi: 10.1016/j.actpsy.2006.03.008

Sokolowski, H. M., Fias, W., Bosah Ononye, C., & Ansari, D. (2017). Are numbers grounded in a general magnitude processing system? A functional neuroimaging meta-analysis. *Neuropsychologia*. doi: 10.1016/j.neuropsychologia.2017.01.019

Stevens, J. C., Mack, J. D., & Stevens, S. S. (1960). Growth of sensation on seven continua as measured by force of handgrip. *Journal of Experimental Psychology*, *59*(1), 60–67. doi: 10.1037/h0040746

van Galen, M. S., & Reitsma, P. (2008). Developing access to number magnitude: A study of the SNARC effect in 7- to 9-year-olds. *Journal of Experimental Child Psychology*, *101*(2), 99–113. doi: 10.1016/j.jecp.2008.05.001

Van Opstal, F., & Verguts, T. (2013). Is there a generalized magnitude system in the brain? Behavioral, neuroimaging, and computational evidence. *Frontiers in Psychology*, *4*, 2011–2013. doi: 10.3389/fpsyg.2013.00435

Xuan, B., Zhang, D., He, S., Chen, X., R., J. S., & F., M. (2007). Larger stimuli are judged to last longer. *Journal of Vision*, *7*(10), 2. doi: 10.1167/7.10.2

Yates, M. J., Loetscher, T., Nicholls, M. E. R., X., C., P., T., & R., M. (2012). A generalized magnitude system for space, time, and quantity? A cautionary note. *Journal of Vision*, *12*(7), 9–9. doi: 10.1167/12.7.9

Zorzi, M., Priftis, K., & Umiltà, C. (2002). Brain damage: Neglect disrupts the mental number line. *Nature*, *417*(6885), 138–139. doi: 10.1038/417138a
