# Supplementary material for: Processing symbolic magnitude information conveyed by number words and by scalar adjectives
Source: Q J Exp Psychol (Hove). 2021 Jul 16;75(3):422–49. doi: 10.1177/17470218211031158 (PMC8793294; doi:10.1177/17470218211031158)
Supplement: sj-docx-2-qjp-10.1177_17470218211031158 – Supplemental material for Processing symbolic magnitude information conveyed by number words and by scalar adjectives [file sj-docx-2-qjp-10.1177_17470218211031158.docx]

**Online Supplementary Material B:
Alternative accounts of the size congruity effect**

One alternative account of the size congruity effect is that participants assign verbal labels to the stimuli that they see on the screen — e.g., labels ‘large’ and ‘small’ are assigned both to digits and physical sizes. The conflict then arises between the verbal labels (e.g., ‘large’ for digit and ‘small’ for physical size in the incongruent condition). If this were the case, we could not conclude that representations of numerical magnitude and physical size are shared based on the size congruity effect. However, there are several reasons to exclude this possibility.

First, if the size congruity effect were purely due to assigning conflicting verbal labels to the stimuli, this conflict should result in the same congruity effect regardless of how large the differences between magnitudes in the irrelevant dimension are. In other words, we would not expect the congruity effect to be modulated by the specific magnitude differences in the irrelevant dimension. However, we know that in addition to the congruity effect, an interaction with the magnitude distance information in the to-be-ignored dimension can be observed in the size-congruity paradigm. Specifically, the size of the congruity effect is modulated by the extent to which two digits differ in the task-irrelevant dimension in terms of the numerical magnitude or in terms of physical font size (referred to as *size* or *numerical distance*; e.g., Arend & Henik, 2015; Cohen Kadosh, Henik, & Rubinsten, 2008; Henik & Tzelgov, 1982; Kaufmann et al., 2005; Santens & Verguts, 2011). Specifically, with a larger difference between the size or numerical magnitude in the task-irrelevant dimension one observes a larger congruity effect (i.e., more interference). This finding shows that the congruity effect is not just driven by the presence of *some* conflicting information; rather the size or strength of this conflicting information matters.

Second, the size congruity effect has also been observed in a similar subliminal priming paradigm where no verbal labeling was possible (Lourenco et al., 2016). Therefore, we conclude that assignment of verbal labels is an unlikely explanation of the size congruity effect.

Another alternative account is based on visual attention capture (Risko et al., 2013). This account is based on the observation from vision research that larger items in a scene capture attention more than small items. Given that in a size congruity paradigm one item is visually larger than the other, this item will capture attention first and, therefore, might have a temporal advantage - it could be processed first. Thus, if the task is to react to the item with the larger numerical magnitude, in the congruent condition the physically larger item is at the same time the target for the numerical magnitude task, and thus target item identification would be boosted (relative to the incongruent condition).

However, this account can only explain the size congruity effect in the numerical comparison task (with size magnitude as the task-irrelevant dimension), whereas the size congruity effect is also observed in the physical comparison task (with numerical magnitude as the task-irrelevant dimension). Specifically, if the congruity effect was explained solely by temporal processing advantage of the larger object on the screen, we would not expect to observe a congruity effect when the participants’ task is to indicate the larger physical size object because then the larger object would be processed first in both conditions. Moreover, as noted by Arend and Henik (2015) this account is meant to explain the size congruity effect when the participants are instructed to choose the numerically larger item, whereas if participants are instructed to choose the numerically smaller item, it predicts a reverse effect - faster responses in the incongruent condition (since attention would still be captured by the larger item first). However, in contrast to this prediction, the size congruity effect is observed with the ‘choose smaller’ instructions too albeit somewhat smaller in effect size (Arend & Henik, 2015; Tzelgov et al., 1992). Arend and Henik (2015) argue that, given the reduction in congruity effect for ‘choose smaller’ instructions, there does seem to be some effect of attention capture in the size congruity task, but it clearly cannot fully explain the congruity effect (a position also accepted as a possibility by Risko et al., 2013 who proposed the account based on visual attention capture). Hence, the attention capturing cannot be taken as the full explanation of the size congruity effect.

**References**

Arend, I., & Henik, A. (2015). Choosing the larger versus choosing the smaller: Asymmetries in the size congruity effect. *Journal of Experimental Psychology: Learning, Memory, and Cognition*, *41*(6), 1821–1830. doi: 10.1037/xlm0000135

Cohen Kadosh, R., Henik, A., & Rubinsten, O. (2008). Are Arabic and verbal numbers processed in different ways? *Journal of Experimental Psychology: Learning, Memory, and Cognition*, *34*(6), 1377–1391. doi: 10.1037/a0013413

Henik, A., & Tzelgov, J. (1982). Is three greater than five: The relation between physical and semantic size in comparison tasks. *Memory & Cognition*, *10*(4), 389–395. doi: 10.3758/BF03202431

Kaufmann, L., Koppelstaetter, F., Delazer, M., Siedentopf, C., Rhomberg, P., Golaszewski, S., . . . Ischebeck, A. (2005). Neural correlates of distance and congruity effects in a numerical Stroop task: An event-related fMRI study. *NeuroImage*, *25*(3), 888–898. doi: 10.1016/j.neuroimage.2004.12.041

Lourenco, S. F., Ayzenberg, V., & Lyu, J. (2016). A general magnitude system in human adults: Evidence from a subliminal priming paradigm. *Cortex*, *81*, 93–103. doi: 10.1016/j.cortex.2016.04.013

Santens, S., & Verguts, T. (2011). The size congruity effect: Is bigger always more? *Cognition*, *118*(1), 94–110. doi: 10.1016/j.cognition.2010.10.014

Risko, E. F., Maloney, E. A., & Fugelsang, J. A. (2013). Paying attention to attention: Evidence for an attentional contribution to the size congruity effect. *Attention, Perception, & Psychophysics*, *75*(6), 1137–1147. doi: 10.3758/s13414-013-0477-2

Tzelgov, J., Meyer, J., & Henik, A. (1992). Automatic and intentional processing of numerical information. *Journal of Experimental Psychology: Learning, Memory, and Cognition*, *18*(1), 166–179. doi: 10.1037/0278-7393.18.1.166
