# Supplementary material for: Processing symbolic magnitude information conveyed by number words and by scalar adjectives
Source: Q J Exp Psychol (Hove). 2021 Jul 16;75(3):422–49. doi: 10.1177/17470218211031158 (PMC8793294; doi:10.1177/17470218211031158)
Supplement: sj-docx-3-qjp-10.1177_17470218211031158 – Supplemental material for Processing symbolic magnitude information conveyed by number words and by scalar adjectives [file sj-docx-3-qjp-10.1177_17470218211031158.docx]

**Online Supplementary Material C:
Key results from the norming studies – RTs and error rates for the selected number word pairs and font size pairs, scalar adjective pairs and font size pairs**

Table 1
*Mean RT (SD) and error rate observed in pre-test for the selected number word pairs and font size pairs.*

| **number word pair** | **RT (SD)** | **error rate** | **font size pair** | **RT (SD)** | **error rate** |
| --- | --- | --- | --- | --- | --- |
| ‘een-zes’ | 701 (135) ms | 0% | 41-47 pt | 720 (222) ms | 2.43% |
| ‘twee-acht’ | 743 (157) ms | 1% | 37-42 pt | 747 (229) ms | 1.74% |
| ‘twee-vijf’ | 780 (202) ms | 0.67% | 41-46 pt | 774 (222) ms | 5.56% |
| ‘drie-acht’ | 790 (170) ms | 3.67% | 38-42 pt | 761 (254) ms | 5.92% |
| ‘vier-acht’ | 810 (180) ms | 1.67% | 43-48 pt | 787 (235) ms | 6.97% |
| across all pairs | 764 (174) ms | 1.4% | across all pairs | 757 (233) ms | 4.52% |

Table 2
*Mean RT (SD) and error rate observed in stimuli pre-test for the selected pairs of scalar adjective and font size combinations.*

| **adjective pair** | **RT (SD)** | **error rate** | **font size pair** | **RT (SD)** | **error rate** |
| --- | --- | --- | --- | --- | --- |
| ‘kort-lang’ | 887 (208) ms | 7% | 43-48 pt | 787 (235) ms | 6.97% |
| ‘laag-hoog’ | 793 (206) ms | 3.83% | 41-47 pt | 720 (222) ms | 2.43% |
| ‘licht-zwaar’ | 834 (229) ms | 0.83% | 37-42 pt | 747 (229) ms | 1.74% |
| ‘dun-dik’ | 829 (217) ms | 5.33% | 38-42 pt | 761 (254) ms | 5.92% |
| ‘stil-luid’ | 884 (246) ms | 4.83% | 41-46 pt | 774 (222) ms | 5.56% |
| across all pairs | 844 (224) ms | 4.36% | across all pairs | 757 (233) ms | 4.52% |
